# Transcriptional profiling reveals roles of intercellular Fgf9 signaling in astrocyte maturation and synaptic refinement during brainstem development

**DOI:** 10.1016/j.jbc.2022.102176

**Published:** 2022-06-23

**Authors:** Ashley N. Brandebura, Douglas R. Kolson, Emily M. Amick, Jad Ramadan, Matthew C. Kersting, Robert H. Nichol, Paul S. Holcomb, Peter H. Mathers, Peter Stoilov, George A. Spirou

**Affiliations:** 1 Graduate Program in Biochemistry and Molecular Biology, West Virginia University, Morgantown, West Virginia, USA; 2 Department of Biochemistry, West Virginia University, Morgantown, West Virginia, USA; 3 Rockefeller Neuroscience Institute, West Virginia University, Morgantown, West Virginia, USA; 4 Department of Otolaryngology HNS, West Virginia University, Morgantown, West Virginia, USA; 5 Department of Ophthalmology, West Virginia University, Morgantown, West Virginia, USA; 6 Department of Medical Engineering, University of South Florida, Tampa, Florida, USA

**Keywords:** development, MNTB, gene regulation, perineuronal nets, Notch pathway, fibroblast growth factor, vascular endothelial growth factor, transforming growth factor beta, angiogenesis, CH, calyx of Held, cKO, conditional KO, CPM, counts per million, DAPI, 4′,6-diamidino-2-phenylindole, FDR, false discovery rate, IFC, integrated fluidic circuit, IHC, immunohistochemistry, LSO, lateral superior olive, MNTB, medial nucleus of the trapezoid body, NA, numerical aperture, PNN, perineuronal net, ROI, region of interest, SBEM, serial block-face electron microscopy, scRNA-Seq, single cell RNA-Seq, smFISH, single molecule FISH, VAC, vascular-associated cell, VCN, ventral cochlear nucleus

## Abstract

Neural tissue maturation is a coordinated process under tight transcriptional control. We previously analyzed the kinetics of gene expression in the medial nucleus of the trapezoid body (MNTB) in the brainstem during the critical postnatal phase of its development. While this work revealed timed execution of transcriptional programs, it was blind to the specific cells where gene expression changes occurred. Here, we utilized single-cell RNA-Seq to determine transcriptional profiles of each major MNTB cell type. We discerned directional signaling patterns between neuronal, glial, and vascular-associated cells for VEGF, TGFβ, and Delta-Notch pathways during a robust period of vascular remodeling in the MNTB. Furthermore, we describe functional outcomes of the disruption of neuron-astrocyte fibroblast growth factor 9 (Fgf9) signaling. We used a conditional KO (cKO) approach to genetically delete *Fgf9* from principal neurons in the MNTB, which led to an early onset of glial fibrillary acidic protein (Gfap) expression in astrocytes. In turn, Fgf9 cKO mice show increased levels of astrocyte-enriched brevican (Bcan), a component of the perineuronal net matrix that ensheaths principal neurons in the MNTB and the large calyx of Held terminal, while levels of the neuron-enriched hyaluronan and proteoglycan link protein 1 (Hapln1) were unchanged. Finally, volumetric analysis of vesicular glutamate transporters 1 and 2 (Vglut1/2), which serves as a proxy for terminal size, revealed an increase in calyx of Held volume in the Fgf9 cKO. Overall, we demonstrate a coordinated neuron-astrocyte Fgf9 signaling network that functions to regulate astrocyte maturation, perineuronal net structure, and synaptic refinement.

The medial nucleus of the trapezoid body (MNTB) in the auditory brainstem is a well-established model system to study neural circuit formation (1, 2). The neuronal population in the rodent MNTB is nearly homogeneous (3, 4, 5), thus eliminating the confounding influence of neuronal diversity on studies of genetic regulation of neural circuit formation in other brain regions. The primary innervation of MNTB has a clear endpoint of monoinnervation by the largest nerve terminal in the mammalian brain, the calyx of Held (CH) (6). Maturation occurs on a compressed timeframe (3, 7, 8, 9), which can be exploited to connect molecular, structural, and electrophysiological events. The CH grows rapidly between postnatal day 2 (P2) and P4 during which the postsynaptic principal neurons increase in size and acquire many mature biophysical properties (3, 7, 8, 9, 10). Over 70% of principal neurons transition from a state of multi-innervation to monoinnervation by P6 (3), after which the extracellular matrix elaborates into distinct perineuronal nets (PNNs) (11, 12, 13). In synchrony with these events, the glial cell population more than doubles between P3 and P6 (5), thereby contributing to the subsequent myelination of globular bushy cell axons that form the CH (14, 15) and development of a close association of astrocyte processes with the CH terminal (16, 17).

Essential steps in neural circuit formation such as axon guidance, synaptogenesis, and synaptic pruning have traditionally been studied in the context of neuron–neuron signaling. More recently, evidence has accumulated suggesting that glial cells play fundamental roles in the regulation of these processes (18, 19). A more global definition of neural circuit formation includes extensive structural changes that occur as the neural tissue matures, such as myelination of axons, angiogenesis and blood–brain–barrier formation, and organization of the extracellular matrix. The contributions of oligodendrocytes, astrocytes, and vascular-associated cells (VACs) to these processes are integral to regulating the speed and fidelity of synaptic transmission and maintenance of neuronal homeostasis (20, 21). Thus, studies on the genetic regulation of neural circuit formation should be undertaken with a whole tissue perspective. Utilizing the temporally constrained and well-defined developmental endpoints of the MNTB as a model system can deepen our understanding of the unique roles that each cell type plays in neural tissue development.

We previously conducted a microarray study across postnatal developmental ages with high temporal resolution on bulk microdissected MNTB tissue (12). Transcripts relevant to axon pathfinding, cell differentiation, cell adhesion, and the extracellular matrix (12) dynamically changed expression levels during a period of rapid CH growth (P3–P4) (3, 8), synaptic refinement (P4–P6) (3), glial cell expansion (P3–P14) (5, 14, 17), and pruning of principal cell projections to target territories (P0–P7 for lateral superior olive) (22). However, the bulk tissue approach did not discriminate between the different cell types present in the tissue. The present work utilizes single cell RNA-Seq (scRNA-Seq) to augment the temporal gene expression profiles with high resolution transcriptome data for each major cell type in the MNTB at P3, a time point coinciding with the initiation of CH growth in mouse (3). We take an unbiased approach to investigating intercellular communication and uncover directional signaling patterns for the vascular endothelial growth factor (VEGF), transforming growth factor β (TGFβ), Delta-Notch, and fibroblast growth factor (FGF) pathways. We demonstrate that structural expansion of the vascular network in the MNTB correlates with TGFβ, VEGF, and Delta-Notch signaling, all prominent pathways connected to angiogenesis (23). Additionally, we utilize a transgenic Notch reporter mouse line (24) to confirm active Delta-Notch signaling in VACs. Furthermore, we show that KO of Fgf9 signaling in the MNTB principal neurons results in an early onset of glial fibrillary acidic protein (Gfap) expression, suggesting that neuronal Fgf9 inhibits expression of Gfap. In addition to effects of neuronal Fgf9 KO on astrocytes, we identify an association between Fgf9 signaling and control of the extracellular matrix and CH terminal size. The MNTB, along with other brain regions such as the spinal cord, deep cerebellar nuclei, and cerebral cortex, is highly enriched for the PNN extracellular matrix structure (25, 26, 27). The protein components of the PNNs in the MNTB have been well characterized at maturity (11), and we previously demonstrated a timeline for the expression of several PNN components during development (12). Now, we define cell type contributions of distinct PNN components using scRNA-Seq data and provide evidence for cell-to-cell communications that coordinate the expression of PNN components. We show that genetic deletion of Fgf9 from principal neurons is accompanied by increased expression of brevican (Bcan), which is astrocyte enriched and an integral component of PNNs, as well as larger CH terminal size. This study provides foundational insights into how neuron–astrocyte FGF signaling has functional impacts on astrocyte maturation, as well as extracellular matrix and synaptic remodeling throughout the nervous system.

## Results

### Hierarchical clustering of single cell transcriptomes identifies all major classes of cells in the early postnatal MNTB

**Figure 1:**
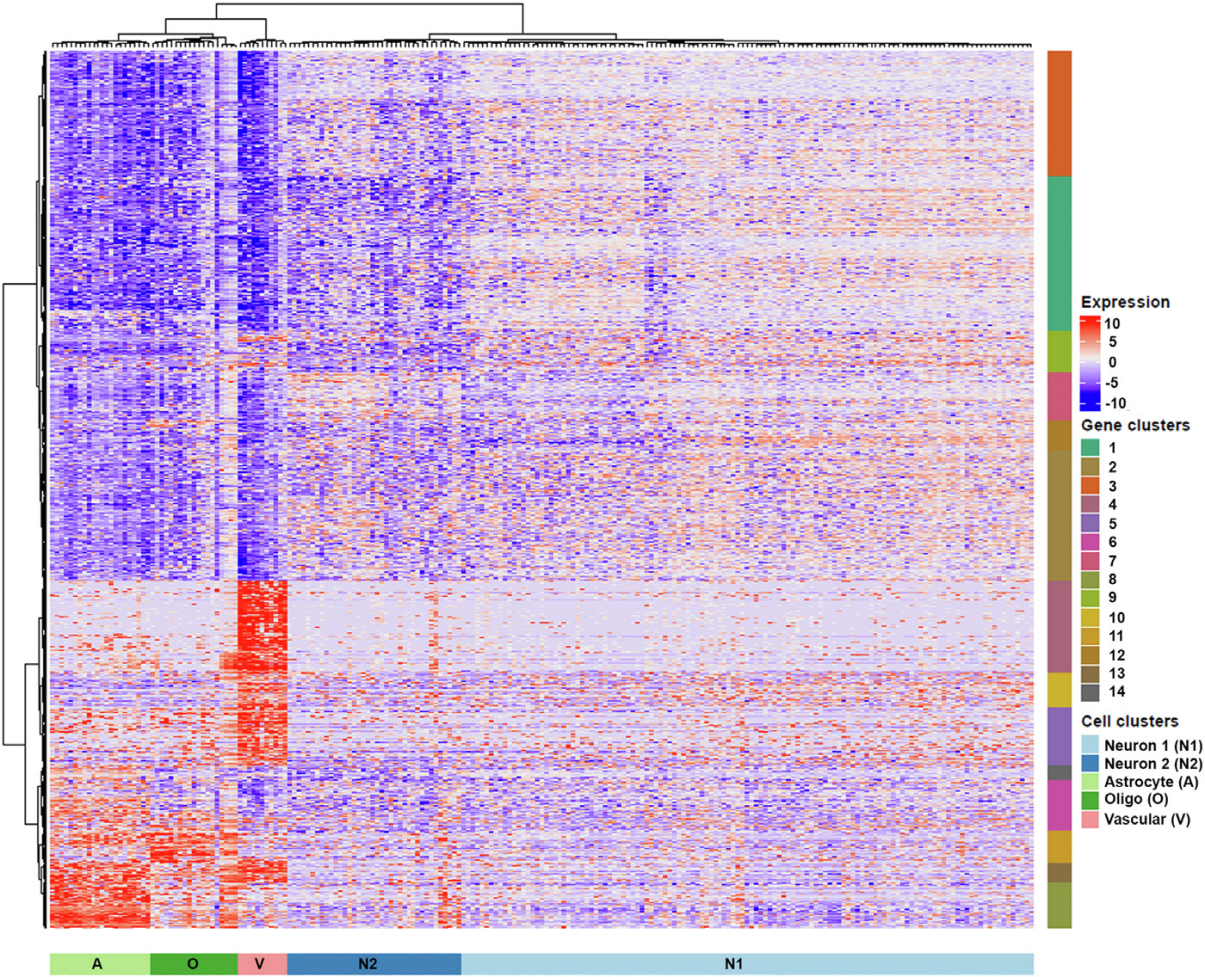
**Hierarchical clustering analysis on cells after sequencing.** Heatmap shows expression levels of genes selected for clustering (*blue* to *red* = low to high expression). Expression values are depicted as the difference from the median in counts per million (CPM) for any particular gene and is displayed on the log_2_ fold change scale. The dendrogram across the *top columns* represents Ward’s distance for each cell (n = 215) and the dendrogram along the side represents Ward’s distance for each gene (n = 1103). The clustering analysis yielded 14 gene clusters (*right side*) and 5 cell clusters (*bottom*) corresponding to two separate neuronal clusters (N1 and N2), an astrocyte cluster (A), an oligodendrocyte cluster (O), and a vascular-associated cell cluster (V).

In order to obtain unbiased transcriptome data for the major cell types in the MNTB, we performed scRNA-Seq analysis on dissociated cells isolated from microdissected MNTB tissue in P3 mice. Cre-positive mice from the *Engrailed1(En1)-Cre;Ai9* cross, which exclusively labels MNTB neurons with tdTomato (4, 5), were utilized so that *tdTomato* transcript levels could function as a validation of the downstream clustering approach (neurons should have the highest *tdTomato* transcript levels). We generated scRNA-Seq libraries from 305 single cells in the MNTB using the Fluidigm C1 system. Libraries from 285 cells were chosen for deep sequencing analysis (Illumina HiSeq instrument; see Experimental procedures) after an initial quality check (Illumina MiSeq instrument; see Experimental procedures) (Fig. S1). After quality control filtering on the deep sequencing data, 215 cells were used for subsequent analysis. The single cell libraries had an average of 8475 genes expressed per cell, with a range of 5804 to 11,207 genes (Fig. S2). Unsupervised hierarchical clustering of these scRNA-Seq data revealed five major transcriptome classes in the MNTB (Fig. 1; neuronal cluster 1; N1; *light blue* n = 125 cells; neuronal cluster 2; N2; *dark blue* n = 38 cells; astrocyte cluster; *light green* n = 22 cells; oligodendrocyte cluster; *dark green* n = 19 cells; VAC cluster; *pink* n = 11 cells).

**Figure 2:**
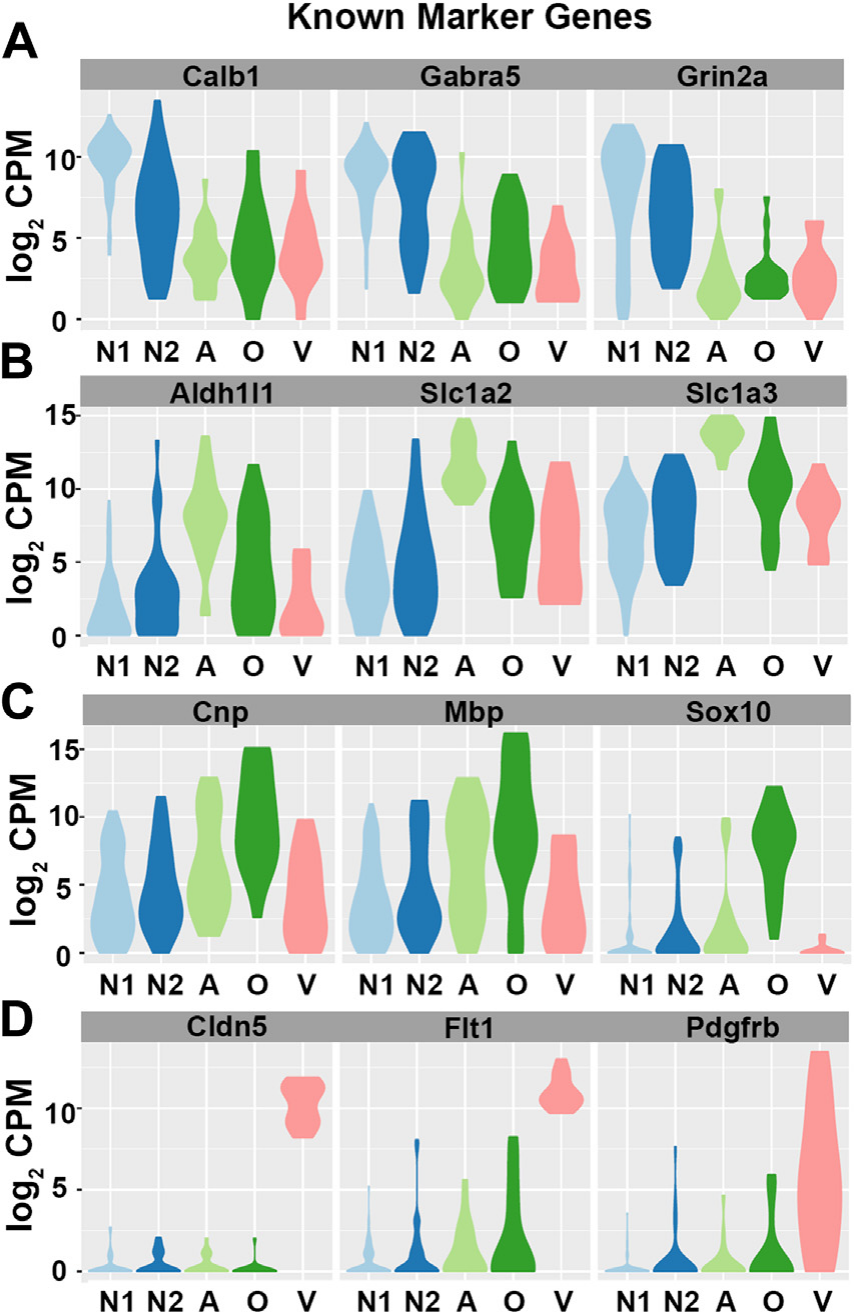
**Identification of cell clusters based on expression of known marker genes.***A*, the height of the violin plots corresponds to expression level in CPM and the width of the violin plot corresponds to the density of cells falling in that expression level. The *light blue* and *dark blue* clusters were identified as neuronal clusters based on the expression of *Calb1*, *Gabra5*, and *Grin2a*. *B*, the *light green* cluster was identified as the astrocyte cluster based on the expression of *Aldh1l1*, *Slc1a2*, and *Slc1a3*. *C*, the *dark green* cluster was identified as the oligodendrocyte cluster based on the expression of *Cnp*, *Mbp*, and *Sox10*. *D*, the *pink cluster* was identified as the VAC cluster based on the expression of *Cldn5*, *Flt1*, and *Pdgfrb*. Expression values are displayed as the log_2_ transformed CPM. N1 = Neuron Cluster 1; N2 = Neuron Cluster 2; A = Astrocyte; O = Oligodendrocyte; V = Vascular Associated Cell. CPM, counts per million; VAC, vascular-associated cell.

After clustering, each group was identified as a specific cell type based on the expression of marker genes. Figure 2 shows the expression levels of three examples of well-established markers for each of the major cell types, including neurons, astrocytes, oligodendrocytes, and VACs (endothelial cells/pericytes combined). The two neuronal clusters (*light blue* and *dark blue*) were identified as neurons using genes known to be expressed in MNTB neurons, including the calcium ion–binding protein (*Calbindin1*; *Calb1*), the GABA receptor subunit (*Gabra5*), and the NMDA receptor subunit (*Grin2a*), among others. MNTB neurons are Calb1-positive by P8 (28) and during development are responsive to NMDA (as well as AMPA) and GABA-mediated currents (8, 29, 30, 31) (Fig. 2*A*). Furthermore, the two neuronal clusters had the highest levels of *tdTomato* transcript, providing a validation of the cell clustering approach (Fig. S6, *p* ≤ 0.005). The astrocyte cluster (*light green*) was identified by expression of the well-known astrocyte marker *aldehyde dehydrogenase family member 1 L1* (*Aldh1L1*) (32). Several reports have demonstrated Aldh1L1 protein expression in MNTB astrocytes at early postnatal ages (5, 12, 17). The transcripts encoding for two other common astrocyte markers, *solute carrier family 1 member 3* (*Slc1a3*; also known as *glial high affinity glutamate transporter 3/Glast*) and *solute carrier family 1 member 2* (*Slc1a2*; also known as *glial high affinity glutamate transporter 2/Glt1*) (33), were also expressed at high levels in the astrocyte cluster (Fig. 2*B*). The oligodendrocyte cluster (*dark green*) was identified based on expression of *SRY-Box 10* (*Sox10*), a common oligodendrocyte marker (34). Sox10 protein was previously shown to be expressed specifically in oligodendrocytes (and not astrocytes) within the MNTB at early postnatal ages (5). Other common oligodendrocyte-associated transcripts that were detected were *2′3′-cyclic nucleotide 3′ phosphodiesterase* (*Cnp*) (35) and *myelin basic protein* (*Mbp*) (36) (Fig. 2*C*). The transcripts that identified the combined endothelial and pericyte cell cluster (*pink*) were *Claudin5* (*Cldn5*, endothelial cell marker) and *FMS-related tyrosine kinase 1* (*Flt1*, endothelial cell marker), along with *platelet derived growth factor receptor β* (*Pdgfrβ*, pericyte marker) (37) (Fig. 2*D*). A microglial cluster was not present, likely because they exist in small numbers at this age (∼1% of total cells in the MNTB) (5) and for technical reasons described in the Discussion section.

### Differential gene expression analysis identified transcripts preferentially expressed in each cell cluster

To identify genes differentially expressed in each cluster, we performed pairwise comparisons of the gene expression levels between the clusters (Tables S1–S10). Genes differentially expressed in each cluster were selected using a twofold differential expression cutoff and false discovery rate (FDR) ≤0.05 based on a previously described scRNA-Seq analysis workflow (38). Notably, only eight genes (*Tbrg1*, *Gm17018*, *Tln2*, *Atp1a2*, *Gstm1*, *Dbi*, *Selenop*, and *Ednrb*; Table S1) were differentially expressed in the pairwise analysis between the two neuronal clusters within the specified significance cut offs (Table S1), reflecting the homogeneous nature of principal neurons in the MNTB. Thus, it is not likely that the two neuronal clusters represent distinct populations of neurons but rather that the clusters are defined by the cumulative effect of relatively moderate differences in the levels of commonly expressed transcripts. In a second round of analysis, each cluster was compared to the average of all other clusters. Especially in the case of the astrocytes and oligodendrocytes, which express many of the same genes, the comparison of one cluster to the average of all other clusters yielded genes highly specific for each cluster. The top 20 transcripts with elevated expression levels in each cluster (cluster 1 and cluster 2 neurons combined, astrocytes, oligodendrocytes, and VACs) are shown in Table 1 and the full lists are shown in Tables S11–S14. The gene sets identified for each cluster correlate well with the assigned cell types further validating our clustering approach (Table 1). Note that the marker genes described previously were differentially expressed but did not always fall within the top 20 differentially expressed genes (*Calb1* for neurons; *Flt1* and *Pdgfrb* for VACs), indicating the robustness of scRNA-Seq to potentially identify new marker genes. Given these gene enrichment patterns by cell type, we next decided to focus on directed intercellular communication within prominent signaling pathways in the scRNA-Seq dataset to determine how these pathways may participate in maturation of the MNTB nucleus.

**Table 1:** Top 20 differentially expressed genes per cell cluster

Neuron				Astrocyte			
Gene name	Fold change	*p*-value	FDR	Gene name	Fold change	*p*-value	FDR
Gm14204	5.33	2.49E-10	1.14E-08	Tnc	8.02	1.09E-12	1.04E-09
**Grin2a**	5.12	4.49E-09	1.59E-07	Fgfr3	6.55	7.53E-09	3.61E-06
C230085N15Rik	5.12	2.61E-08	7.58E-07	9330159F19Rik	4.80	8.43E-07	2.02E-04
Gm42616	4.67	5.25E-08	1.48E-06	Gm3764	4.20	1.24E-06	2.27E-04
Ass1	4.43	1.12E-07	3.00E-06	Aqp4	5.03	1.77E-06	2.27E-04
Ankrd45	4.79	1.23E-07	3.19E-06	Slc6a11	4.90	1.83E-06	2.27E-04
Tmem59l	4.21	1.34E-07	3.37E-06	Tmem229a	5.76	1.90E-06	2.27E-04
Cda	5.11	2.05E-07	5.05E-06	Agt	5.85	3.58E-06	3.44E-04
Syngr3	3.92	3.36E-07	7.74E-06	**Slc1a3**	3.93	1.28E-05	8.83E-04
Lamp5	3.46	6.15E-07	1.31E-05	Fads2	5.31	1.33E-05	8.83E-04
Ramp3	4.32	1.75E-06	3.29E-05	AI464131	5.61	1.38E-05	8.83E-04
Epb41l4b	4.19	5.01E-06	8.43E-05	Pla2g7	4.47	1.78E-05	1.06E-03
Cds1	3.92	6.92E-06	1.13E-04	**Slc1a2**	4.41	1.87E-05	1.06E-03
Rasgrf1	4.48	8.11E-06	1.27E-04	Acot1	5.85	2.17E-05	1.10E-03
**Gabra5**	3.63	8.65E-06	1.30E-04	Kxd1	4.75	2.56E-05	1.19E-03
Car10	3.37	1.01E-05	1.46E-04	Igsf11	5.47	2.61E-05	1.19E-03
Fsd1	4.04	1.03E-05	1.47E-04	Hepacam	4.46	3.70E-05	1.61E-03
Kcnk1	4.13	1.47E-05	2.01E-04	Tulp4	3.89	3.98E-05	1.62E-03
Hapln1	3.00	1.57E-05	2.12E-04	**Aldh1l1**	4.86	4.04E-05	1.62E-03
Prepl	4.14	1.62E-05	2.14E-04	Adgrg1	4.19	6.52E-05	2.40E-03


Oligodendrocyte				Vascular Associated Cells			
Bcas1	9.02	2.81E-12	2.70E-09	Acvrl1	12.13	1.51E-44	1.45E-41
Ralgps2	6.18	5.59E-10	2.68E-07	Il2rg	11.17	2.99E-38	1.43E-35
Ugt8a	8.31	1.70E-09	5.15E-07	Ifitm3	11.40	8.59E-36	2.75E-33
Shisal1	8.17	2.15E-09	5.15E-07	**Cldn5**	12.49	3.30E-33	7.91E-31
Tnr	6.82	1.17E-08	2.23E-06	Ctla2a	12.24	3.05E-32	5.84E-30
Gpr17	6.60	6.72E-08	1.07E-05	Srgn	11.78	2.48E-31	3.97E-29
Plp1	6.09	1.14E-07	1.56E-05	Icam2	11.30	5.48E-31	7.51E-29
Scrg1	6.82	1.56E-07	1.78E-05	Eng	11.32	3.40E-29	4.08E-27
**Mbp**	6.15	1.79E-07	1.78E-05	Grap	11.52	6.68E-27	7.12E-25
**Sox10**	7.35	1.86E-07	1.78E-05	Robo4	10.80	1.10E-26	1.05E-24
Cyfip2	4.75	4.00E-07	3.48E-05	Pltp	11.61	2.60E-22	2.26E-20
Olig2	6.15	8.75E-07	6.99E-05	Slc38a5	11.24	1.17E-21	9.39E-20
**Cnp**	5.58	1.09E-06	8.08E-05	Cd34	10.46	5.04E-21	3.72E-19
Dscam	4.82	1.38E-06	9.45E-05	Tgfbr2	11.03	5.63E-21	3.86E-19
Sema5a	5.47	1.96E-05	9.38E-04	Fzd6	9.57	1.80E-20	1.15E-18
Sox6	5.36	2.16E-05	9.57E-04	Tek	10.16	7.58E-20	4.54E-18
Nfasc	4.14	4.54E-05	1.67E-03	Adgrl4	10.57	1.13E-19	6.36E-18
Rnd2	4.29	5.52E-05	1.83E-03	Lama4	8.99	2.21E-19	1.18E-17
Opcml	4.60	6.05E-05	1.87E-03	Cgnl1	10.20	6.60E-19	3.33E-17
Omg	4.86	1.38E-04	3.40E-03	Adgre5	9.87	2.65E-18	1.27E-16

The top 20 genes differentially expressed in the neuron (N1 and N2 combined) cluster, astrocyte cluster, oligodendrocyte cluster, and VAC cluster are listed in order sorted by lowest FDR. The gene name with fold change (log_2_), *p*-value and FDR are listed. The calculations of differential gene expression are based on the average CPM values of one cluster compared to the average CPM values of all other clusters. FDR ≤0.05 with ≥2-fold change. The marker gene names from Figure 2 that fell within the top 20 differentially expressed genes for each cluster are bolded in the table (*Grin2a* and *Gabra5* for neurons; *Aldh1l1*, *Slc1a2*, and *Slc1a3* for astrocytes; *Cnp*, *Mbp*, and *Sox10* for oligodendrocytes; *Cldn5 for VACs*).

### VEGF, TGFβ, and Delta-Notch signaling pathways are temporally linked to expansion of the MNTB vascular network

The VEGF pathway consists of five ligands and three receptor tyrosine kinases. VEGF signaling mediates vasculogenesis, the generation of the vasculature, as well as angiogenesis, the formation of new vessels from existing vessels (23). The neonatal brainstem increases in volume during development and presumably must expand its vascular network existing at birth. Consistent with models of angiogenesis in other tissues, receptor transcripts (*Flt1* and *Kdr* [also called *VEGFR1* and *VEGFR2*]) were detected at significantly higher expression levels in the VAC cluster compared to the average of all clusters combined (*Flt1* eightfold higher expression; *p*-value ≤ 0.001; *Kdr* 7.9-fold higher expression; *p*-value ≤ 0.001; Table S14 and Fig. S7*A*). *Flt4*, also called *VEGFR3*, was detected at elevated levels in the VAC cluster, although it was not statistically significant. Transcripts for the VEGF ligands, *Vegfa* and *Vegfb*, were detected at moderate levels in all cell clusters, with *Vegfb* showing some selectivity for nonvascular cells. *Vegfc* was detected at lower levels than the other ligands but was more selective for the oligodendrocyte and VAC clusters (Fig. S7*A*). These data suggest that neurons and glial cells participate in secretion of VEGF ligands that signal through receptors in the VACs (Fig. S7*C*).

Like VEGF signaling, TGFβ signaling is also involved in vascular remodeling (23). The TGFβ superfamily encompasses a large set of ligands, which includes TGFβs, BMPs, activins (inhibins as antagonists), growth and differentiation factors (GDFs), and Nodal, among others (39). These ligands signal through a complex of type I and type II receptors. The type III receptor has no signaling domain and instead functions to present ligand and promote complex formation between type I and type II receptors (40). Two receptor transcripts (*Acvrl1* and *Tgfbr2*) and a coreceptor transcript (*Eng*) were significantly differentially expressed in the VAC cluster (*Acvrl1* 12.1-fold higher expression, *p*-value ≤ 0.001; *Tgfbr2* 11.0-fold higher expression, *p*-value ≤ 0.001; *Eng* 11.3-fold higher expression, *p*-value ≤ 0.001; Table S14 and Fig. S7*B*). *Tgfbr3* and *Acvr2a* receptor transcripts were also expressed at the highest levels in the VAC cluster (Fig. S7*B*). The inhibin ligands (dimers of *Inha* and *Inhba/Inhbb* combinations) and activin ligands (dimers of *Inhba* and *Inhbb* combinations) were detected at consistently low levels across cell types. *Gdf11*, another TGFβ-related ligand, was expressed by all cell clusters along with the transcripts for its cognate receptor set (*Acvr1b*, *Tgfbr1*, and *Acvr2a*; Fig. S7*B*). Expression of *Gdf11* and its receptors across all cell types confounds interpretation of directionality in this signal. Among the classical TGFβ ligands, *Tgfb2* was expressed at high levels and *Tgfb3* at low levels across all cell types (Fig. S7*B*). These data suggest that while all cell types are capable of producing TGFβ receptor ligands, primarily TGFβ2, VACs are likely the only cell type that transduces the signal (Fig. S7*C*).

Delta-Notch signaling also plays an important role in maintenance of vascular progenitor cell populations, cell fate determination, and vascular remodeling in the nervous system (23). The Jagged (Jag) and Delta-like (Dll) ligands transduce signaling by binding to four Notch receptors (41). The scRNA-Seq data revealed that each of the *Notch1-3* receptor transcripts were expressed in the astrocyte, oligodendrocyte, and VAC clusters but not the neuronal clusters (Fig. S8*A*). All three transcripts had significantly higher expression in the astrocyte cluster compared to the average of all other clusters (*Notch1* 3.7-fold higher expression; *p*-value ≤ 0.005, *Notch2* 4.3-fold higher expression; *p*-value ≤ 0.001; *Notch3* 3.9-fold higher expression; *p*-value ≤ 0.005; Table S12 and Fig. S8*A*), whereas the VAC cluster contained the highest transcript levels of the *Notch4* receptor transcript (Fig. S8*A*). Delta-Notch signaling regulates tip *versus* stalk cell fate determination for vascular remodeling (23). It was previously demonstrated that the *Dll4* ligand in tip cells transduces active Notch signal to nearby stalk cells to inhibit sprouting, whereas Jag1 expression in stalk cells promotes sprouting angiogenesis through the Notch receptors in tip cells (42). In our scRNA-Seq data we observed a bimodal expression pattern in the VAC cluster violin plots where a smaller number of VACs express high levels of Dll4 and a comparably larger number of VACs express high levels of *Jag1* (Fig. S8*A*). These data may indicate Delta-Notch regulation of sprouting angiogenesis in the MNTB. A readout of active Delta-Notch signaling is upregulation of *Hes1* transcript. The astrocyte and VAC clusters had elevated levels of *Hes1* (Fig. S8*A*), suggesting active Notch signaling in these cell types. Transcripts for the Dll and Jag ligands were detected at the highest levels in the oligodendrocyte and VAC clusters (*Dll1* in oligodendrocyte cluster, *Dll4* in VAC cluster, *Jag1* in oligodendrocyte, and VAC clusters; Fig. S8*A*). These data suggest activation of the Delta-Notch pathway primarily in astrocytes and VACs by Dll and Jag ligands presented by the oligodendrocytes and VACs (Fig. S8*B*).

**Figure 3:**
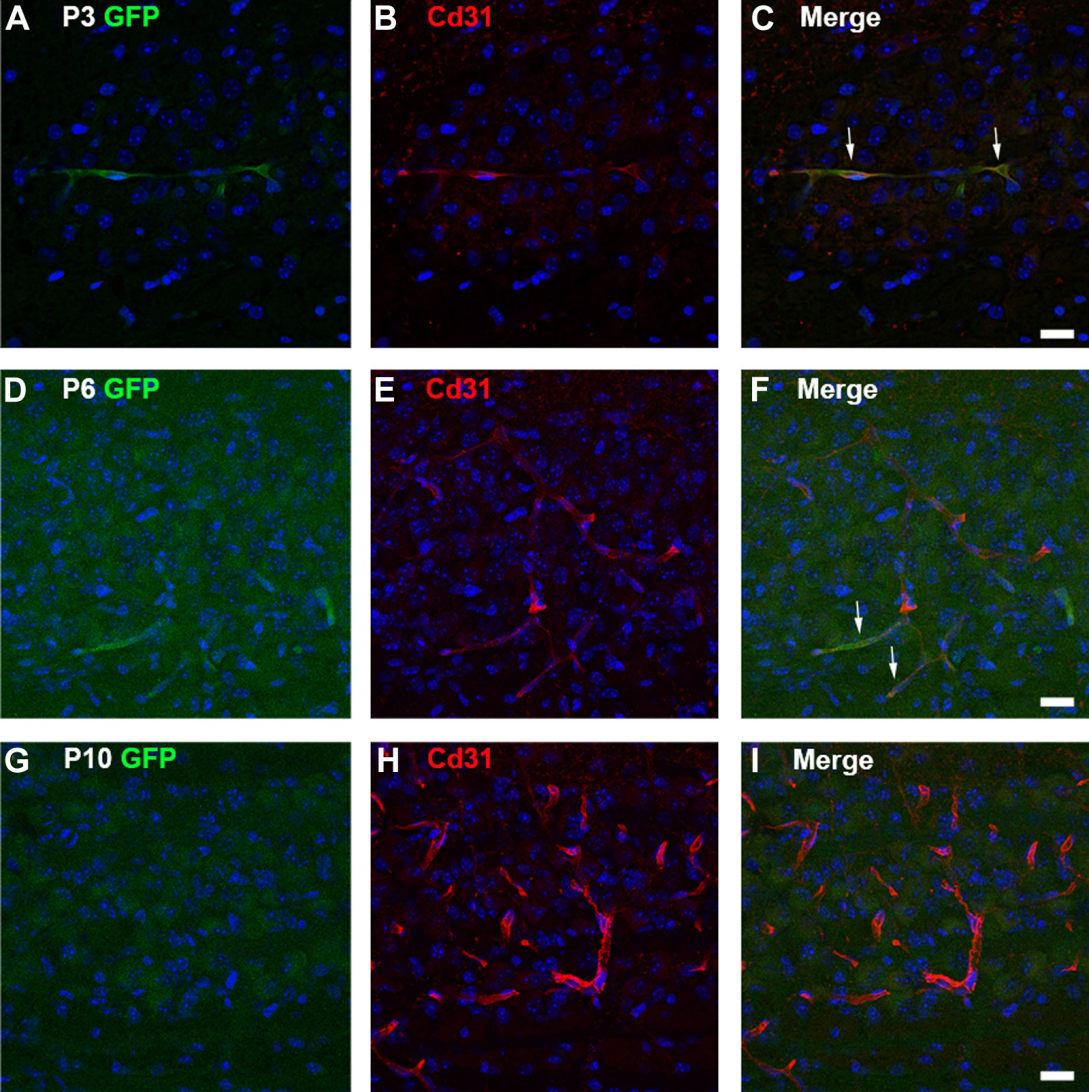
**Delta-Notch signaling active in VACs.***A*, the transgenic Notch reporter mouse was used to demonstrate colocalization of the GFP reporter with (*B* and *C*) the endothelial cell marker Cd31 at P3. *White arrows* denote areas of colocalization. *D*, at P6 the GFP reporter signal is dim and just above background level but (*E* and *F*) does colocalize with Cd31 (*white arrows*). *G*–*I*, at P10 the GFP reporter signal is absent from Cd31+ cells. The scale bars represent 20 μm. VAC, vascular-associated cell.

In order to investigate if Delta-Notch signaling was indeed active in the developing MNTB and in which cell type, we utilized a transgenic Notch reporter mouse, in which GFP expression reports active Delta-Notch signaling (24). GFP reporter expression colocalized with the vascular endothelial cell marker, Cd31 (also known as Pecam, (43)), at P3 (Fig. 3, *A*–*C*). The reporter signal was dimmer at P6 but could still be observed in Cd31+ cells (Fig. 3, *D*–*F*). By P10 the GFP reporter signal could not be discerned above background level (Fig. 3, *G*–*I*). Quantification of GFP signal demonstrated a significant reduction in the percentage of GFP+ area in the MNTB from P3 to P10 (mean at P3 = 1.1 ± 0.34%, mean at P6 = 0.93 ± 1.1%, mean at P10 = 0.0 ± 0.0%, q-value = 0.82 for P3 *versus* P6, ∗q-value = 0.0052 for P3 *versus* P10, q-value = 0.21 for P6 *versus* P10, multiple *t* tests with Benjamini, Krieger, and Yekutieli correction, q = 1%; Fig. S9), demonstrating a transient activation of Delta-Notch signal transduction in the VAC population. GFP labeling in astrocytes was not observed, despite high levels of Hes1 transcript (Fig. S8*A*).

**Figure 4:**
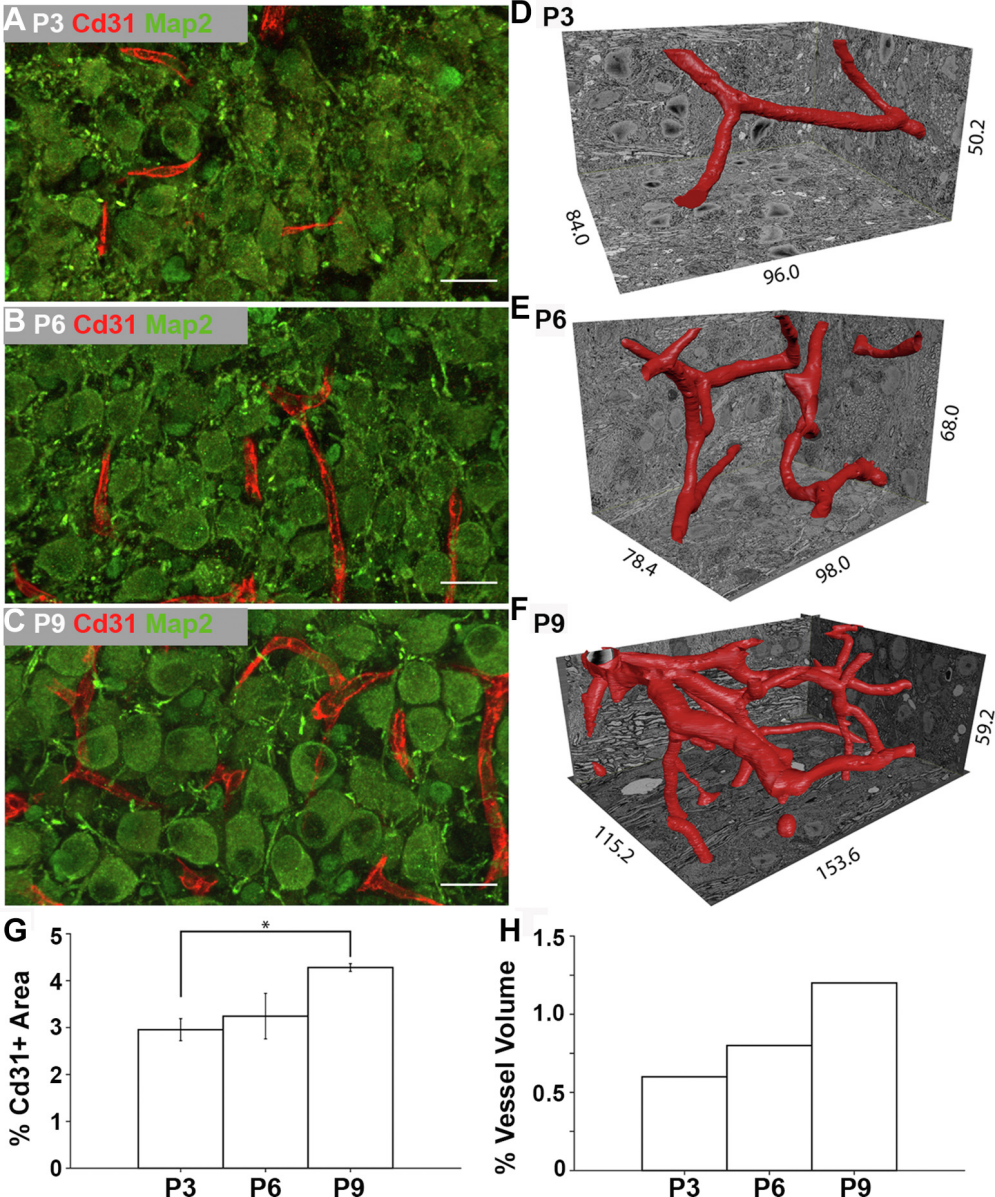
**Angiogenesis in MNTB.***A*–*C*, representative images of the endothelial cell tight junction protein Cd31 (*red*) and Map2-positive neurons (*green*) in the MNTB at P3 (*A*), P6 (*B*), and P9 (*C*). The scale bars represent 20 μm. *D*–*F*, the blood vessels from the P3 (*D*), P6 (*E*), and P9 (*F*) SBEM volumes were segmented and reconstructed as 3D objects (P3 dimensions: x = 96.0 μm, y = 84.0 μum, z = 50.2 μm; P6 dimensions: x= 98.0 μm, y = 78.4 μm, z = 68.0 μm; P9 dimensions: x = 153.6 μm, y = 115.2 μm, z = 59.2 μm). *G*, the percentage of Cd31-positive pixels out of total pixels in the MNTB significantly increases from P3 to P9 (q-value = 0.41 for P3 *versus* P6, ∗∗q-value = 0.00078 for P3 *versus* P9, q-value = 0.022 for P6 *versus* P9, multiple *t*-tests with Benjamini, Krieger, and Yekutieli correction, q = 1% n = 3 at each age). Error bars represent SD. *H*, the percentage of electron microscopy tissue volume occupied by blood vessels increases across development from 0.6% at P3 to 0.8% at P6 to 1.2% at P9. n = 1 at each age. MNTB, medial nucleus of the trapezoid body.

Because of the prominence of three signaling pathways related to vascular remodeling in the sequencing data, we investigated further if the presence of VEGF, TGFβ, and Delta-Notch pathway transcripts is linked to structural remodeling of the vasculature. In support of the VEGF, TGFβ, and Delta-Notch pathways promoting angiogenesis in the MNTB, *Nestin* transcript, a marker of neovascularization in endothelial cell progenitors in early postnatal development (44), was expressed at significantly higher levels in the VAC cluster (4.7-fold higher expression; *p*-value ≤ 0.001; Table S14). In order to check for vascular remodeling at the protein level, antibody labeling for Cd31 was used to determine if there is an expansion of the endothelial cell population across MNTB development. Cd31-positive staining was quantified within the MNTB at P3, P6, and P9 and normalized for the size of the MNTB. Cd31 staining represents 3.0 ± 0.2% of the MNTB at P3 and 3.2 ± 0.5% at P6. At P9 there is a significant increase in Cd31-positive staining to 4.3 ± 0.1% of the MNTB (q-value = 0.41 for P3 *versus* P6, ∗∗q-value = 0.00078 for P3 *versus* P9, q-value = 0.022 for P6 *versus* P9, multiple *t* tests with Benjamini, Krieger, and Yekutieli correction, q = 1%; Fig. 4, *A*–*C* and *G*). However, the increase in Cd31-positive staining could be due to a developmental upregulation of Cd31 protein. To verify that there is structural expansion of the vasculature occurring, blood vessels were segmented and reconstructed from three-dimensional electron microscopy volumes at P3, P6, and P9 time points (Fig. 4, *D*–*F*). The percent occupancy of serial block-face electron microscopy (SBEM) volume of the blood vessels was calculated. Blood vessels represented 0.6% of the SBEM volume at P3 and 0.8% of the volume at P6. Concordant with the progressive increase in Cd31 labeling, there is doubling of the blood vessel volume to 1.2% of the SBEM volume at P9 compared to P3 (Fig. 4*H*). Taken together, these data indicate that VEGF, TGFβ, and Delta-Notch signaling precede a structural expansion of the vasculature in the developing MNTB. These data provide an example of how static transcriptional data can be used to predict dynamic structural changes in a developing neural tissue.

### FGF signaling components are differentially expressed in neurons and astrocytes

**Figure 5:**
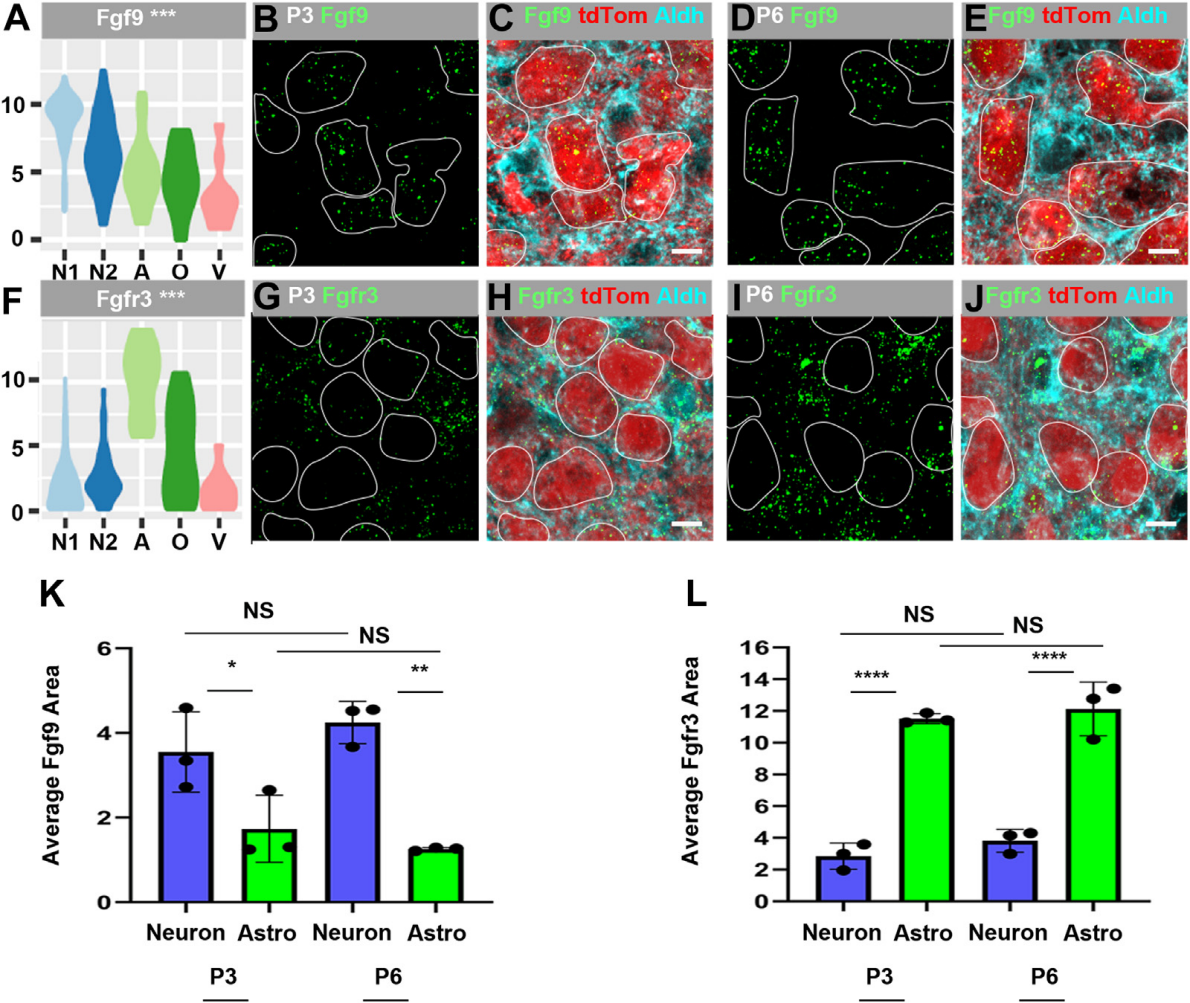
**Fgf9 ligand is expressed in MNTB neurons and Fgfr3 is expressed in MNTB astrocytes.***A*, *Fgf9* is significantly differentially expressed in the neuronal clusters at P3. *B* and *D*, *Fgf9* (*green*) colocalizes with (*C* and *E*) tdTomato-positive neurons (*red* outlined in *white*) in the *Engrailed1-Cre;Ai9* cross at P3 and P6. *F*, *Fgfr3* is significantly differentially expressed in the astrocyte cluster at P3. *G* and *I*, *Fgfr3* (*green*) is present outside of (*H* and *J*) tdTomato-positive neuronal cell bodies (outlined in *white*) and largely colocalizes with Aldh1L1-positive astrocytes (*cyan*). *K*, quantification of *Fgf9* probe area within MNTB neurons shows similar expression levels at P3 and P6 and enrichment in neurons compared to astrocytes (mean of 3.55% ± 1.0% at P3 in neurons and 4.25% ± 0.5% at P6 in neurons; mean of 1.7% ± 0.8% at P3 in astrocytes and 1.3% ± 0.04% at P6 in astrocytes, two-way ANOVA: age [F(1,8) = 0.080, *p* = 0.79], cell type [F(1,8) = 38.92, *p* = 0.0002], interaction [F(1,8) = 2.32, *p* = 0.17], post-hoc Tukey’s: ∗*p* ≤ 0.05 neurons *versus* astrocytes at P3 and ∗∗*p* ≤ 0.01 neurons *versus* astrocytes at P6, n = 3 independent experiments). *L*, quantification of *Fgfr3* probe area shows similar expression levels in astrocytes at P3 and P6 and enrichment in astrocytes compared to neurons (mean of 11.5% ± 0.3% at P3 in astrocytes and 12.1% ± 1.8% at P6 in astrocytes, mean of 2.8% ± 0.8% at P3 in neurons and 3.8% ± 0.7% at P6 in neurons, 2-way ANOVA: age [F(1,8) = 1.79, *p* = 0.22], cell type [F(1,8) = 206.8, *p* ≤ 0.0001], interaction [F(1,8) = 0.094, *p* = 0.77], post-hoc Tukey’s: *p* ≤ 0.0001 astrocytes *versus* neurons at P3 and P6, n = 3 independent experiments). The scale bars represent 10 μm. Error bars represent SD. MNTB, medial nucleus of the trapezoid body.

Components of the FGF signaling pathway were also prominent in the sequencing dataset. FGF signaling plays important roles in organ development, including the brain, by mediating the growth, differentiation, and fate of different cell types. The FGF family is comprised of 18 secreted FGF ligands, four intracellular FGF proteins, and four tyrosine kinase receptors (45). Transcripts encoding for five secreted FGF ligands (*Fgf1*, *Fgf2*, *Fgf9*, *Fgf10*, and *Fgf18*) and three FGF receptors (*Fgfr1*, *Fgfr2*, and *Fgfr3*) were detected in the MNTB (Fig. S10*A*). Of the Fgf ligands, *Fgf2* exhibited low expression, while *Fgf1*, *Fgf10*, and *Fgf18* exhibited comparatively higher expression levels distributed amongst all cell types. The Fgf9 transcript was detected at significantly higher levels in the neuronal cluster compared to the average of the other clusters (3.3-fold higher expression; *p*-value ≤ 0.001; Table S11 and Fig. 5*A*). *Fgf9* has high affinity for Fgfr2 and Fgfr3 (46). The *Fgfr3* transcript was differentially expressed and detected at a relatively high level, in the astrocyte cluster (6.6-fold higher expression; *p*-value ≤ 0.001; Table S12 and Fig. 5*F*).

We performed single molecule FISH (smFISH) to validate the *Fgf9* and *Fgfr3* expression patterns. *Fgf9* colocalizes with tdTomato-positive neurons in the *En1-Cre;Ai9* cross at P3 (cell bodies outlined in white in Fig. 5, *B* and *C*) and P6 (cell bodies outlined in Fig. 5, *D* and *E*). In contrast, *Fgfr3* can visually be seen to overlay with Aldh1L1-positive astrocyte cell bodies at P3 (cyan in Fig. 5, *G* and *H*) and P6 (*cyan* in Fig. 5, *I* and *J*). Quantification of probe area was performed to determine if levels of *Fgf9* and *Fgfr3* change between ages and to investigate enrichment patterns of ligand and receptor between cell types. *Fgf9* probe area in MNTB neurons revealed similar levels of *Fgf9* in neurons at P3 and P6 and enrichment of *Fgf9* in neurons compared to astrocytes at both ages (Fig. 5*K*; mean of 3.55% ± 1.0% at P3 in neurons and 4.25% ± 0.5% at P6 in neurons, *p* > 0.05; mean of 1.7% ± 0.8% at P3 in astrocytes and 1.3% ± 0.04% at P6 in astrocytes, ∗*p* ≤ 0.05 neurons *versus* astrocytes at P3 and ∗∗*p* ≤ 0.01 neurons *versus* astrocytes at P6, two-way ANOVA, n = 3 independent experiments). Quantification of *Fgfr3* probe area in MNTB astrocytes reveals similar levels of transcript at P3 and P6 and enrichment in astrocytes compared to neurons at both ages (Fig. 5*L*; mean of 11.5% ± 0.3% at P3 in astrocytes and 12.1% ± 1.8% at P6 in astrocytes, *p* > 0.05; mean of 2.8% ± 0.8% at P3 in neurons and 3.8% ± 0.7% at P6 in neurons, *p* ≤ 0.0001 astrocytes *versus* neurons at P3 and P6, two-way ANOVA, n = 3 independent experiments). Thus, the smFISH expression patterns for *Fgf9* and *Fgfr3* match the scRNA-Seq data (Fig. S10*A*), providing further confidence in the scRNA-Seq predictions for cell type–specific expression patterns. These data suggest secretion of FGF ligands primarily by neurons and relatively specific signaling by neuronal Fgf9 to astrocytes *via* the Fgfr3 receptor (Fig. S10*B*).

### Neuron-astrocyte Fgf9 signaling controls onset of GFAP expression in astrocytes

**Figure 6:**
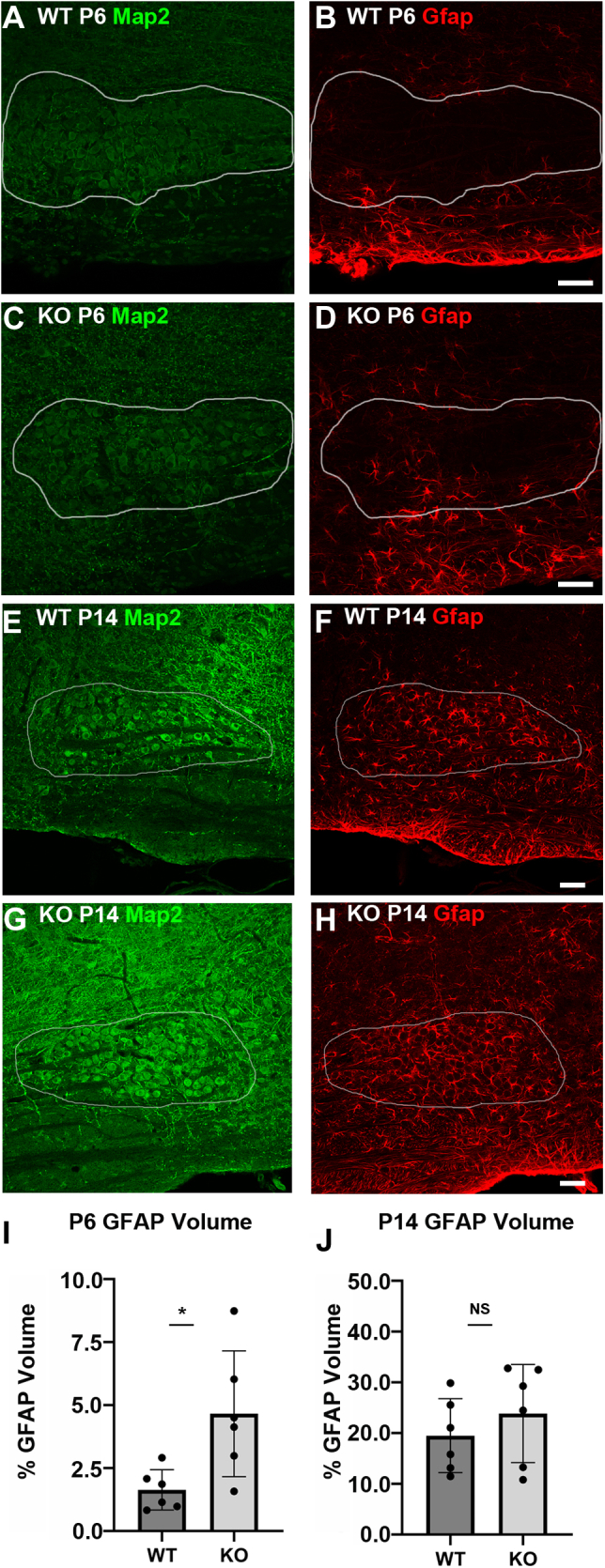
**Genetic deletion of principal neuron Fgf9 results in early astrocytic expression of Gfap.***A*–*D*, Fgf9 cKO mice display an increased level of Gfap staining within the boundaries of the MNTB nucleus at P6 compared to WT littermates. The *white outlines* represent the boundaries of the MNTB based on Map2 staining. The scale bars represent 50 μm. *E*–*H*, at P14, the Fgf9 cKO mice display similar levels of Gfap staining within the MNTB compared to WT littermates. The *white outlines* represent the boundaries of the MNTB based on Map2 staining. The scale bars represent 50 μm. *I*, quantification of Gfap volume normalized to MNTB volume at P6 reveals a significant increase in Gfap levels in the cKO mice (percent volume of MNTB in WT = 1.6 ± 0.8% and percent volume of MNTB in cKO = 4.7 ± 2.5%, *p* ≤ 0.05, n = 6, unpaired *t* test). *J*, at P14 WT and Fgf9 cKO mice have similar levels of Gfap expression in the MNTB (percent volume of MNTB in WT = 19.4 ± 7.3% and percent volume of MNTB in cKO = 23.8 ± 9.7% *p* ≥ 0.05, n = 6, unpaired *t* test). Error bars represent SD. cKO, conditional KO; MNTB, medial nucleus of the trapezoid body.

Next, we asked if neuron-astrocyte Fgf9 signaling has functional impact on the development of MNTB astrocytes. We utilized a cross of the *En1-Cre* mouse line and a floxed Fgf9 line to generate a conditional KO of Fgf9 (hereafter referred to as Fgf9 cKO) in MNTB neurons. A previous *in vitro* study demonstrated Fgf9 treatment inhibited the differentiation of fate-unspecified adult neural progenitor cells into differentiated astrocytes and caused an almost complete blockade of Gfap expression (47). Therefore, we decided to investigate if genetic deletion of Fgf9 from MNTB neurons *in vivo* had downstream effects on Gfap expression in astrocytes. During normal MNTB development, astrocytes within the boundaries of the nucleus have only very minimal Gfap reactivity at P6 and Gfap protein levels subsequently increase by ∼P14 (17). Using immunostaining at P6 and P14, we find that astrocytes within the MNTB nucleus of Fgf9 cKO mice develop an early onset of Gfap reactivity (Fig. 6, *A*–*D*). The volume of Gfap signal within the MNTB is significantly increased in cKO mice compared to their WT littermates (Fig. 6*I*, percent volume of Gfap label in MNTB in WT = 1.6% and percent volume of MNTB in cKO = 4.7%, *p* ≤ 0.05, n = 6, unpaired *t* test). By P14 the volume of Gfap within the MNTB is similar between the genotypes (Fig. 6, *E*–*H* and *J*, percent volume of MNTB in WT = 19.4% and percent volume of MNTB in cKO = 23.8%, *p* ≥ 0.05, n = 6, unpaired *t* test). The early onset of Gfap expression in MNTB astrocytes could indicate an accelerated maturation period. As a control to ensure the increase in Gfap expression at P6 was not due to increased proliferation and/or migration of astrocytes into the MNTB, we performed cell counts using Aldh1L1 antibody to label the cell bodies of astrocytes. There was no difference in the percentage of astrocytes out of total cells in the MNTB at P6 (Fig. S11*A*, WT = 22.9% ± 3.1% and cKO = 21.7% ± 2.4%, *p* > 0.05, n = 3, unpaired *t* test). Additionally, we quantified Gfap levels in a neighboring anatomical nucleus, the lateral superior olive (LSO), where *Engrailed1* is not expressed (3) and there should not be Cre recombination. There were no differences in Gfap levels in the LSO (Fig. S11*B*, percent volume of Gfap label WT = 0.28% ± 0.2% and cKO = 0.32% ± 0.2%, *p* > 0.05, n =3, unpaired *t* test).

### Reduced Fgf9 signal transduction to astrocytes is accompanied by changes to PNN structure and CH terminal size

Given the intimate association of astrocyte processes with the PNN extracellular matrix that ensheaths the CH and the principal neurons in the MNTB (11), as well as physical interaction of astrocyte processes with the CH itself (16), we next investigated if PNN and CH structures were altered in the Fgf9 cKO mice. First, we used the scRNA-Seq data at P3 to provide insight into each cell type’s contribution to the PNN structure at later ages and identify candidate markers to measure in the Fgf9 cKO mice. Many PNN-associated transcripts were detected as differentially expressed between the cell clusters (Fig. S12*A* and Tables S11–S14). Among these transcripts were *Hapln1* in neurons (threefold higher expression; *p*-value ≤ 0.001), *Bcan* in astrocytes and oligodendrocytes (3.4-fold higher expression; *p*-value ≤ 0.005), *Ncan* in astrocytes (3.2-fold higher expression; *p*-value ≤ 0.001), *Vcan* in astrocytes (3.5-fold higher expression; *p* ≤ 0.005), and *Tnr* in oligodendrocytes (6.8-fold higher expression; *p*-value ≤ 0.001). Other transcripts detected but not differentially expressed were *Acan*, *Hapln3*, and *Hapln4*, where *Acan* had highest expression levels in neurons, *Hapln3* in astrocytes and VACs, and *Hapln4* in neurons and astrocytes (Fig. S12*A*). The scRNA-Seq data now allow us to broadly characterize the expression patterns of different PNN components across cell types, where neurons have the highest levels of *Hapln* transcripts, astrocytes and oligodendrocytes have the highest levels of *Tnr* and the chondroitin sulfate proteoglycan transcripts (excluding *Acan*, which has higher levels in neurons), and VACs have relatively lower levels of all PNN-associated transcripts (Fig. S12*B*).

**Figure 7:**
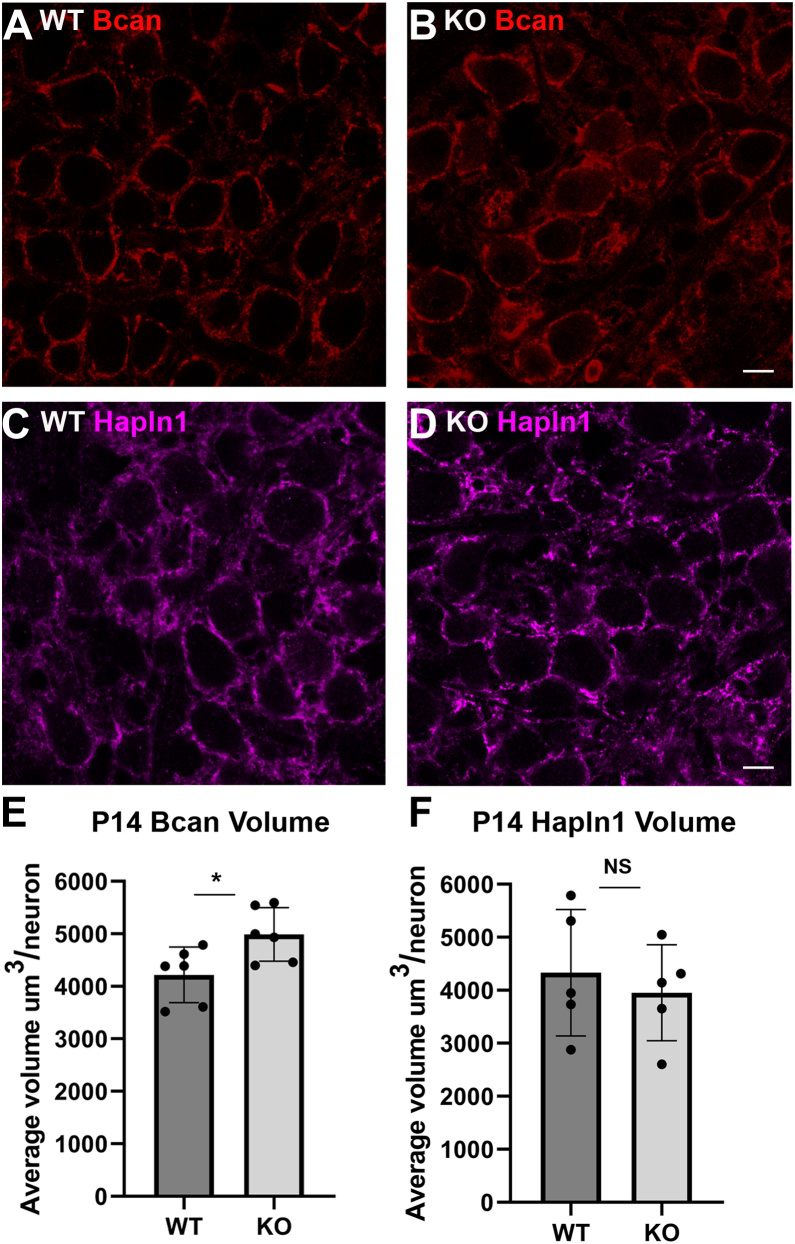
**Fgf9 cKO mice have increased Bcan volume in the PNN.***A* and *B*, Bcan staining (*red*) is increased in Fgf9 cKO mice at P14 compared to WT littermates and displays a staining pattern that is less interdigitated. *C* and *D*, Hapln1 staining (*magenta*) appears similar between WT and KO mice. The scale bars represent 10 μm. *E*, quantification of Bcan volume surrounding the principal neurons reveals a significant increase in Fgf9 cKO mice (mean volume of WT = 4216 ± 529.4 μm^3^ and mean volume of cKO = 4986 ± 510.3 μm^3^, *p* ≤ 0.05, n = 6, unpaired *t* test). *F*, volumetric analysis shows no significant change in Hapln1 volumes between genotypes (mean volume of WT = 4330 ± 1192.4 μm^3^ and mean volume of cKO = 4952 ± 905.4 μm^3^, *p* ≥ 0.05, n = 5, unpaired *t* test). Each point on the graph represents the average of all quantified Bcan- or Hapln1-containing nets/replicate. Sixteen nets were quantified/replicate. Error bars represent SD. cKO, conditional KO; PNN, perineuronal net.

In order to investigate a potential correlative effect on the early Gfap reactivity in astrocytes and changes in PNN structure, we investigated Bcan and Hapln1 immunolabeling by performing volumetric analysis on these components in the Fgf9 cKO mice. *Bcan*, *Ncan*, and *Vcan* transcript levels were enriched in astrocytes in the scRNA-Seq dataset (Fig. S12*A*). Bcan and Ncan, but not Vcan, protein can be reliably detected in MNTB at mature ages, but only Bcan shows an early developmental pattern of expression that temporally correlates with CH growth and refinement to monoinnervation (11, 12, 13). Therefore, Bcan was chosen for analysis. Conversely, *Hapln1* was enriched in neurons in the sequencing data (Fig. S12*A*), and we previously demonstrated Hapln1 protein expression in the MNTB at early postnatal ages (12). Our results demonstrate differences in the volume of Bcan between genotypes, where the Fgf9 cKO mice displayed significantly larger volumes of Bcan in the PNN structure compared to WT littermates (mean volume of WT = 4216 μm^3^ and mean volume of cKO = 4986 μm^3^, *p* ≤ 0.05, n = 6, unpaired *t* test) and appear to have a fuller, less interdigitated staining pattern (Fig. 7, *A*, *B* and *E*). In order to discern whether PNNs are larger in general, or if specific protein components of the PNNs are affected, we also performed volumetric analysis of neuron-enriched Hapln1 at the same time point. Interestingly, the volume of Hapln1 staining did not change in the cKO compared to the WT (Fig. 7, *C*, *D* and *F*; mean volume of WT = 4330 μm^3^ and mean volume of cKO = 4952 μm^3^, *p* ≥ 0.05, n = 5, unpaired *t* test). The specific change in the astrocyte-enriched Bcan levels suggests that normal Fgf9 signaling through astrocytes may negatively regulate the deposition of Bcan in the PNN structure, similar to the Fgf9 effects on Gfap labeling.

**Figure 8:**
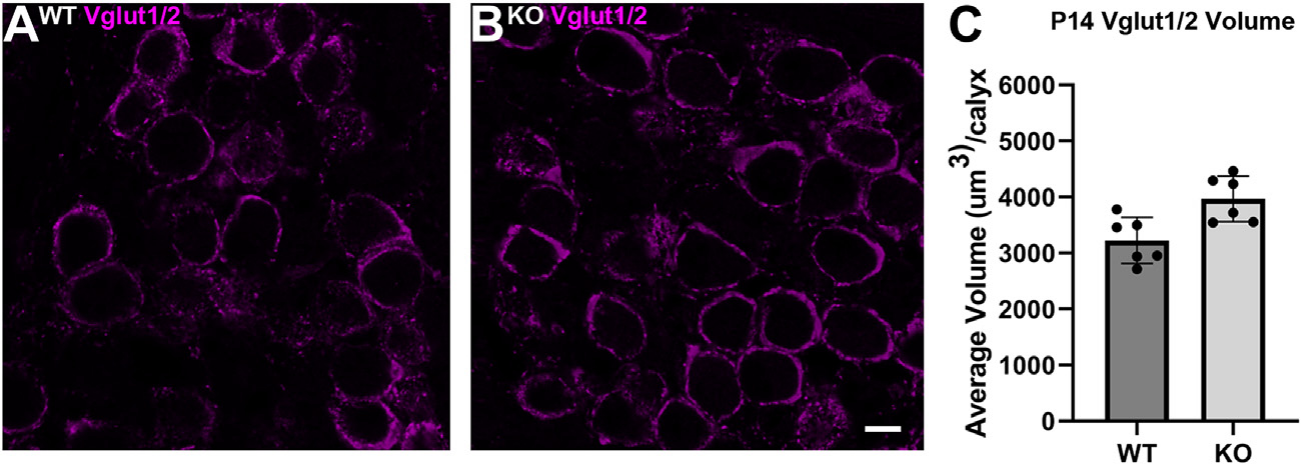
**Fgf9 cKO mice have increased Vglut1/2 volume in the CH.***A* and *B*, Vglut1/2 staining (*magenta*) is increased in Fgf9 cKO mice at P14 compared to WT littermates. The scale bar represents 10 μm. *C*, quantification of Vglut1/2 volume within the CH shows a significant increase in Fgf9 cKO mice (mean volume of WT = 3224 ± 412.8 μm^3^ and mean volume of cKO = 3966 ± 408.3 μm^3^, *p* ≤ 0.05, n = 6, unpaired *t* test). Each point on the graph represents the average of all quantified calyces/replicate. Sixteen calyces were quantified/replicate for a total of 96 calyces. Error bars represent SD. CH, calyx of Held; cKO, conditional KO.

PNNs enwrap the CH terminal, and Bcan KO mice display altered physiology at the CH terminal, including increased action potential transmission delay and duration of presynaptic and postsynaptic action potentials, as well as decreased vesicular glutamate transporter 1 (Vglut1) signal in the presynaptic terminal (48). Therefore, we hypothesized that the increased Bcan volume may alter the size of the CH. We used double staining for Vglut1 and Vglut2 as a readout for CH terminal size. Volumetric quantification demonstrated that Fgf9 cKO mice have significantly larger Vglut1/2 volumes compared to their WT littermates (Fig. 8, *A*–*F*, mean volume of WT = 3224 μm^3^ and mean volume of cKO = 3966 μm^3^, *p* ≤ 0.05, n = 6, unpaired *t* test). Therefore, Bcan in the PNN and CH terminal size increase in parallel in the Fgf9 cKO, implicating a role for Fgf9 signaling in negative regulation of PNN size and synaptic terminal size.

## Discussion

This study is the first to report cell type–specific transcriptional profiles in the MNTB. This brain region has proven to be an informative model system to study neural circuit formation due to the homogeneity of neuronal cell type, rapid synchronized formation of synaptic contacts, and presence of a well-defined extracellular matrix (7, 8, 10, 11). scRNA-Seq analysis yielded five major transcriptional clusters corresponding to two clusters of neurons and one each of astrocytes, oligodendrocytes and VACs. From these expression profiles, we determined that VEGF, TGFβ, and Delta-Notch signaling are correlated with structural expansion of the vasculature during MNTB tissue maturation. We identified FGF signaling as a prominent pathway in the transcriptional dataset and investigated the functional outcomes of genetic deletion of the Fgf9 ligand from MNTB neurons during development. The cKO of Fgf9 restricts gene deletion effects to the MNTB, without potential signals relayed from upstream cell groups (cochlea and ventral cochlear nucleus [VCN]) that would be affected by a generalized KO. Through this investigation, we now define a neuron-astrocyte FGF signaling network that regulates astrocyte maturation, PNN structure, and the size of the CH terminal in the MNTB.

### Technical approach to investigate cell type–specific gene expression in the MNTB

Transcriptional studies performed at multiple developmental time points are useful in elucidating dynamic changes in gene expression level and how these might relate to defined maturational events in the system. Multiple previous studies used microarrays to investigate developmental transcriptional dynamics in various regions of the auditory pathway, including the VCN (49), whole superior olivary complex (50), and microdissected MNTB (12). These studies have been instrumental in identifying transcripts that dynamically change across development but have masked the individual cell types to which these transcripts belong due to the use of bulk tissue samples.

Cell type–specific transcriptional studies are necessary to understand transcriptional regulation in each individual cell type. Körber *et al.* (51) were the first to perform a neuron-specific transcriptional study in the VCN using a laser capture microdissection approach, where they were able to identify transcripts changing before and after the onset of hearing. Recently, scRNA-Seq technology was used to characterize subclasses of spiral ganglion neurons in the auditory nerve (52, 53) and to identify rare cell populations in the cochlea (54). In this study, we performed scRNA-Seq in the MNTB at a single time point (P3) at the onset of CH growth to generate a comprehensive database of cell type–specific transcriptional profiles for neurons, glia, and VACs. We previously showed that commonly used Cre lines for astrocytes (*Aldh1L1-Cre*) and oligodendrocytes (*PDGFRα-Cre*) were not restrictive enough in the MNTB to be useful for specific cell isolation (5). scRNA-Seq proved to be a powerful tool that allowed us to transcriptionally characterize the major cell types in the MNTB without relying on reporters for isolation. Additionally, the scRNA-Seq data can now be used in a complementary manner with our temporal microarray dataset (12) to identify genes that are both cell type–specific and significantly change across MNTB development.

The scRNA-Seq approach in the MNTB was strengthened by the use of mice from the *En1-Cre;Ai9* cross. In previous studies, *En1-Cre;Ai9* mice were demonstrated to exclusively label most neurons in the MNTB (97% of neurons and 0.0% of Aldh1L1+, Sox10+, and Iba1+ cells) (4, 5). The correlation of high levels of tdTomato transcript in cells that fell into neuronal clusters validates the clustering approach. Although limited tdTomato transcript was detected in other cell groups (Fig. S6), we attribute this to slight leakage of the highly expressed tdTomato transcript in the microfluidics capture device. After cell capture and prior to lysis, each capture site was imaged for tdTomato fluorescence and annotated as a probable neuron if tdTomato+. There were not occurrences of error in the cell clustering based on tdTomato fluorescence. We argue that any tdTomato transcript detected in outlier cells from non-neuronal clusters did not alter the clustering results or differential gene expression analysis. We performed single cell capture using the largest microfluidic plate size available (17–25 μm) for the Fluidigm C1 platform because the microfluidics in smaller plates were plugged by the large MNTB neurons. However, the large plate size biases against capture of smaller cells (glial cells and VACs). For this reason, the relative percentages of each cell type captured do not exactly mirror the actual percentages of cell types present in the MNTB at P3 (5). We were unable to profile microglial cells because they only represent 1% of the total cells in the MNTB at P3 (5) and their small size biased against their capture in the microfluidics plate size we utilized.

### Distinct neuronal clusters may represent slight differences in synaptic activity levels, maturation, or position along MNTB tonotopic axis

In contrast with other species (cats, bats, and guinea pigs), to our knowledge there are no reports of nonprincipal cells in the mouse MNTB (55, 56, 57). The homogeneous nature of MNTB neurons is represented in the scRNA-Seq data, where the two neuronal clusters only have eight differentially expressed genes using stringent cut offs. It is unlikely that the two neuronal clusters represent contamination with cells from outside the MNTB borders. The MNTB microdissection approach (12) carefully excludes the extreme lateral boundaries of the MNTB. It is noteworthy that *Calb1* transcript was present in both neuronal clusters, and neurons from the neighboring superior paraolivary nucleus are Calb1 negative (28), arguing that sampling was contained within the borders of the MNTB. Cells from each of the six independent cell capture batches were present in both neuronal clusters, ruling out batch effects due to technical variation or slight differences in age. Instead, we speculate that the two neuronal clusters may represent a developmental shift related to major maturational events that occur in the MNTB during the first postnatal week. These include growth of bouton-sized terminals into large CH terminals, strengthening and pruning of principal neuron projections to the LSO, and establishment of the tonotopic axis.

At P3 about one-half (∼43%) of principal neurons receive at least one large terminal and others receive only small-sized inputs, which can partly explain slight differences in transcript levels between the two neuron groups. Argininosuccinate synthase (Ass1) is a critical enzyme in the cycle that produces nitric oxide (NO) (58), and MNTB neurons produce NO in response to increased synaptic activity (59). Higher levels of *Ass1* transcript in neuronal cluster 1 may be reflective of differences in activity levels related to the growing size of the presynaptic terminal inputs. Likewise, *Akap5* transcript, which encodes for a scaffolding protein in the postsynaptic density involved in AMPA receptor recruitment (60), was also found at higher levels in neuronal cluster 1. Secondly, maturation of the MNTB:LSO projections may also be reflected in the two neuronal clusters. Transcripts such as *Vsnl1*, *Atp6v1g2*, *Rab3b*, *Snap25*, *Nrxn1*, and *Cask* are related to presynaptic vesicle release and/or presynaptic cell adhesion and protein scaffolding and were all higher in neuronal cluster 1, which may represent the strengthening and pruning of MNTB:LSO projections occurring during the first postnatal week (22). The MNTB is tonotopically organized, and in many aspects, the high frequency region matures faster than the low frequency region (61). The sequencing of MNTB was performed at P3 before establishment of tonotopy but subtle transcriptional differences may be detectable earlier than physiological differences. The potential correlation of upregulation of specific transcripts related to metabolism and synaptic transmission with larger CH terminals and/or refinement of MNTB target innervations and tonotopy will require further investigation. Future studies could utilize electrophysiological recordings and cell labeling in the MNTB followed by sequencing of the patched or stimulated MNTB cells to correlate level of synaptic activity, axon branching, and transcript expression levels.

### Transcriptional data maps cell-to-cell signaling networks in the developing MNTB

In this study, we identified several examples of signaling pathways transcriptionally active during MNTB development that have known involvement in angiogenesis and vascular remodeling, including VEGF, TGFβ, and Delta-Notch signaling (23, 62). Transcripts encoding for the receptors of the TGFβ and VEGF pathways were most highly expressed in the VAC cluster, and ligands were expressed in the neuronal and glial cell clusters. The transcript expression patterns for Delta-Notch signaling components suggested tripartite signaling involving oligodendrocytes, VACs, and astrocytes. A transgenic Notch reporter mouse (24) allowed us to define which cell types have functionally active canonical Delta-Notch signaling. We found evidence for developmentally regulated Delta-Notch signaling in Cd31+ vascular endothelial cells (Fig. 3). Violin plots show a smaller number of Dll4-high expressing cells than Jag1-high expressing cells (Fig. S8*A*), which is in agreement with a previously described model whereby Dll4 ligand in the fewer tip cells transduces active Notch signal to nearby more numerous stalk cells to inhibit sprouting and Jag1 expression in stalk cells promotes sprouting angiogenesis through the Notch receptors in tip cells (42). These data together with structural demonstration of vascular expansion (Fig. 4) nicely demonstrate how the scRNA-Seq approach across multiple cell types can relate cell signaling interactions to the total transformation of tissue structure that defines early brain development.

We were surprised by a lack of GFP reporter expression in the astrocyte population, despite enrichment of several components of Delta-Notch signaling in the astrocyte cluster (Fig. S8*A*). Several studies have demonstrated Delta-Notch signaling in cortical astrocytes (32, 37) including functional studies showing that Delta-Notch signaling promotes an astrocyte cell fate from radial glial cells and subsequent astrocyte differentiation (63). The lack of signal from the GFP reporter in astrocytes could be due to their developmental stage, noncanonical Delta-Notch signaling, or technical issues with the reporter such as low signal levels or cell type differences in the transgene expression.

### A neuron to astrocyte Fgf9 signaling axis modulates astrocyte maturation, PNN structure, and synaptic refinement at the CH

The scRNA-Seq data identified significant differential expression of *Fgf9* in neurons and its high-affinity receptor, *Fgfr3*, in astrocytes. We validated these findings using smFISH and demonstrated these enrichment patterns are maintained at P6 (Fig. 5), when the CH terminal achieves its large size and monoinnervation endpoint onto most MNTB neurons (3). A previous *in vitro* study demonstrated that application of Fgf9 to neurosphere cultures strongly inhibited a transition to a Gfap+ astrocyte cell fate (47). Our work now demonstrates that Fgf9 dosage modulates the timing of Gfap acquisition in MNTB astrocytes *in vivo*. Loss of Fgf9 leads to an aberrant early onset of Gfap expression (with no change in astrocyte numbers within the MNTB), indicating an accelerated maturation of the astrocytes and the normal inhibitory role of Fgf9 in this process.

The early onset maturation of astrocytes was accompanied by a parallel increase in Bcan volume. Although we cannot directly link early astrocyte maturation to increased Bcan in the PNNs, our scRNA-Seq data show enrichment of *Bcan* transcripts in astrocytes compared to other cell types in the MNTB (Fig. S12*A*). Increased Bcan may have physiological consequences on synaptic transmission at the CH, as Bcan KO mice display increased delay of synaptic transmission and longer duration of presynaptic and postsynaptic action potential waves forms in the MNTB (48).

Astrocyte processes are in contact with the growing CH terminal (17), and they substantially cover the MNTB neuronal cell body until P6 *via* thin, vellus processes (3). It is likely, then, that astrocytes modulate CH growth and maturation and may do so by limiting their contact with the postsynaptic neuron. Along with the increase in Bcan volume, we also observed an increase in CH volume in the Fgf9 cKO, which may reflect early astrocyte activation and removal of vellus processes from the MNTB neuron surface. Further study using extensive volume electron microscopy, which reveals the vellus processes, could reveal these physical changes in astrocytes in the Fgf9 cKO. Bcan immunolabeling appears adult-like in level and cellular distribution at P14 relative to P6-7 (12, 13), in parallel with the onset of hearing and morphological fenestration of the presynaptic membrane (64). Bcan KO mice have similar CH volume as assayed by tract tracing and Vglut2 labeling but have less Vglut1 labeling (48). Our combination of Vglut1 and 2 circumvents effects on individual transporters and suggests a connection between early astrocyte activation and perhaps astrocyte-derived PNN components and CH terminal size. PNNs have a strong association with restriction of plasticity. For example, enzymatic digestion of PNNs in the visual cortex results in enhanced ocular dominance plasticity that normally does not exist after closure of the visual critical period in development (65) and digestion of PNNs in the spinal cord promotes axonal sprouting after injury (66). In this case, accelerated astrocyte maturation in the MNTB could remove limitations to growth of the CH terminal and drive increased Bcan secretion, leading to early closure of synaptic plasticity that cements the larger size and limits pruning of the CH terminal. In line with this idea, Ribot *et al.* (67) recently demonstrated in visual cortex that astrocytes directly participate in closure of the critical period of plasticity through inhibition of a matrix-degrading enzyme that occurs sequentially after astrocytes begin to express the gap junction protein, Connexin30. Thus, astrocyte participation in construction of the PNNs after downregulation of Fgf9 signaling may be a similar mechanism to promote mature circuit wiring and limit plasticity.

In this study, we assign cell type–specificity to transcripts encoding for ligands and receptors of the VEGF, TGFβ, Delta-Notch, and FGF signaling pathways and provide a valuable resource for the scientific community to utilize for probing cell–cell signaling interactions in neural circuit formation. We further provide functional data demonstrating that perturbations of neuron-astrocyte Fgf9 signaling result in early astrocyte maturation that is paralleled by alterations to PNN structure and synaptic refinement at the CH. These findings emphasize that neural circuit formation in early development requires tissue transformation involving all cell types and provide a framework for future investigations of direct roles for astrocytes in PNN development and synaptic refinement that may be generalized to other brain regions.

## Experimental procedures

All procedures involving animals were approved by the West Virginia University Institutional Animal Care and Use Committee.

### Animal breeding

*Engrailed1-Cre* (*En1-Cre*) heterozygous male mice (68), which were a generous gift from Dr Mark Lewandoski, were crossed to a Cre-dependent tdTomato reporter line (*Ai9*; stock no. 007905, Jackson Laboratory; homozygous females) (69). Cre-positive mouse pups aged P3 from the *En1-Cre;Ai9* cross were used for all scRNA-Seq and smFISH experiments. The *En1-Cre;Ai9* reporter cross was previously validated to specifically label neuronal cells in the MNTB with high efficiency (4, 5, 70). Transgenic Notch enhanced GFP reporter mice (Jax #018322, (24)) were used to assay active Delta-Notch signaling. Immunohistochemistry (IHC) experiments were performed using WT C57Bl6/J mice (Jackson Laboratory, stock no. 000664). The SBEM volumes were generated from FVB/NJ mice. MNTB principal neuron Fgf9 cKO mice were generated by crossing heterozygous *En1-Cre* mice to homozygous *Fgf9 floxed/floxed* mice (Jax # 030675; (71)). Then *En1-Cre+*;*Fgf9 floxed* hemizygous mice were crossed again to homozygous *Fgf9 floxed/flox*ed mice to generate a full KO. In all instances, P0 is defined as the day of birth.

### MNTB tissue collection and dissociation

*En1-Cre*-positive (thus tdTomato fluorescence-positive) mice from the *En1-Cre;Ai9* cross were used at P3 for the scRNA-Seq experiments. MNTB tissue from at least three mice per litter was pooled together, and in total six litters were used for six separate cell capture procedures. The MNTB tissue was microdissected as described previously (12). Briefly, brains were removed from the skull in ice-cold low Ca^2+^ artificial cerebrospinal fluid containing 125 mM NaCl, 2.5 mM KCl, 3 mM MgCl_2_, 0.1 mM CaCl_2_, 25 mM glucose, 25 mM NaHCO_3_, 1.25 mM NaH_2_PO_4_, 0.4 mM ascorbic acid, 3 mM myo-inositol, and 2 mM Na-pyruvate (all chemicals obtained from Millipore Sigma) and saturated with 95% O_2_/5% CO_2_. Coronal sections of brainstem containing MNTB were sliced at 200 μm on a VF-200 Compresstome (Precisionary Instruments Inc). Slices that contained MNTB on both sides were placed into ice-cold artificial cerebrospinal fluid solution, and the MNTB was microdissected from the surrounding tissue using a 26-gaude needle under a dissecting microscope.

Microdissected MNTB tissue was dissociated in Earl’s balanced salt solution with papain (20 U/ml) and DNase (95 U/ml; Papain Dissociation System, Worthington Biochemical Corporation) at 37 °C for 7 min. In the last minute of dissociation, the mixture was gently mechanically triturated with a fire-polished glass Pasteur pipette (Thermo Fisher Scientific). An inhibitor solution (Papain Dissociation System, Worthington Biochemical Corporation) containing Earl’s balanced salt solution with bovine serum albumin (10 mg/ml), ovomucoid protease inhibitor (10 mg/ml), and DNase (9.5 U/ml) was added to halt further enzymatic dissociation. The solution was then centrifuged at 500*g* for 10 min at 4 °C (accuSpin Micro 17R, Thermo Fisher Scientific). After removing the supernatant, the pellet was resuspended in wash buffer containing Hank’s balanced salt solution with 1.5 mg/ml glucose (Thermo Fisher Scientific) and 0.1% fraction V bovine serum albumin (Millipore Sigma). Two additional rounds of washing were performed on the cell suspension alternating with centrifugation (500*g* for 10 min at 4 °C) and resuspension. Before the final wash, the cell suspension was passed through a 30 μm mesh filter (Sysmex Partec GmbH) to remove cell clumps and debris. Following the final centrifugation, the pellet was resuspended in a mixture containing 60% wash buffer and 40% C1 suspension reagent (C1 Single Cell Reagent Kit for mRNA Seq, Fluidigm).

### Single cell microfluidics capture and preparation of complementary DNA

Single cells were captured and lysed using the C1 microfluidics-based system (Single Cell Auto-Prep System, Fluidigm) with the standard protocol provided by the manufacturer (PN 100-7168 K1). A total of six integrated fluidic circuits (IFCs; size 17–25 μm, Fluidigm) were used to capture cells from six separate litters of *En1-Cre;Ai9* mice (genotype Cre-positive) at age P3. Briefly, the C1 IFC was primed using the mRNA Seq: Prime 1773x script as provided by the manufacturer. After priming, the single cell suspension was loaded into the IFC. Additionally, a 10 μM calcein AM (Thermo Fisher Scientific) solution in Hank’s balanced salt solution was added into inlet 1 of the IFC to assess cell viability. Cells were captured and stained using the mRNA Seq: Cell Load & Stain 1773x script. Each capture site of the IFC was then imaged on an EVOS Fl Manual microscope (Thermo Fisher Scientific) using brightfield, Texas Red (for tdTomato), and GFP (for calcein AM) filter cubes. Dead cells (calcein AM-negative), doublets, and empty capture sites were marked for exclusion from library preparation in the downstream workflow. tdTomato-positive cells were denoted as probable neurons based on expression of the fluorescent protein driven by Cre-mediated recombination from the *En1* promoter, which was previously demonstrated to label ∼97% of neurons in the MNTB (5). After imaging, the IFC was returned to the C1 to run the mRNA Seq: RT and Amp 1773x script. The workflow used the SMARTer Ultra Low RNA Kit (Clontech) and the Advantage 2 PCR Kit (Clontech) for lysis, complementary DNA synthesis, and PCR amplification. RNA spikes (ArrayControl spikes #1/4/7, Thermo Fisher Scientific) were added in the lysis mix in a linear range of concentrations to check fidelity of PCR amplification. After PCR amplification (21 rounds), the product was collected from the C1 IFC and transferred into a 96-well plate with C1 DNA dilution reagent (C1 Single Cell Reagent Kit for mRNA Seq, Fluidigm) and stored at −20 °C until library preparation.

### Library preparation

The concentration of each complementary DNA sample was measured using a Qubit 2.0 fluorometer and dsDNA high sensitivity reagent (Thermo Fisher Scientific). Each library was diluted to 0.2 ng/μl optimally with C1 harvest reagent (C1 Single Cell Reagent Kit for mRNA Seq, Fluidigm) but libraries were used if they were at least 0.1 ng/μl. Library preparation and indexing were performed using the Nextera XT Library Preparation Kit and the Nextera XT 96 Index Kit v2 (Illumina), according to the manufacturer’s instructions. Single cell libraries were pooled together and purified using AMPure XP beads (Beckman Coulter) at 90% of total pool volume. The samples were eluted from the beads with C1 DNA dilution reagent. The concentration and library size distribution of each sample was measured using an Agilent 2100 Bioanalyzer (Agilent Technologies).

### Sequencing

Equal volumes of each library were added to the pool for initial sequencing on an Illumina MiSeq instrument. Alignment was performed to GRCm38 using HiSat2 (72) with spike and tdTomato transcript sequences added to the reference file. The number of usable reads was calculated by subtracting the number of reads aligned to spike sequences from the total number of reads. It was noted that the probable neurons (tdTomato-positive during imaging described previously) had a lower proportion of reads aligning to spike sequences than the other cell types. Therefore, the MiSeq step was useful to adjust the final volumes of each library added to the final pool for deep sequencing, allowing for a more equal representation of endogenous reads in each library. Paired-end 75 base pair deep sequencing was performed at an average depth of 3.1 million reads per library on the Illumina HiSeq 4000 at the Roy J. Carver Biotechnology Center (University of Illinois at Urbana-Champaign).

### Analysis of sequencing data

Features were counted using Rsubread (73). A previously described scRNA-Seq analysis workflow (38) was used as a template with slight modifications. The workflow utilized the scater, scran, and edger packages in R Studio (RStudio, version 3.4.3) (38, 74, 75). Poor quality cell libraries were removed using two metrics: number of expressed genes and percent of reads aligning to mitochondrial genes. Histograms were generated for all cell libraries for “Number of Expressed Genes” (Fig. S1*A*) and “Mitochondrial Proportion (%)” (Fig. S1*B*). A cutoff of 3 mean absolute deviations (mad; ≤/≥3 mad for number of genes expressed; ≥3 mad for percent of mitochondrial reads) was used to filter for outliers (38). The same metrics after filtering for outliers are displayed in Fig. S1, *C* and *D*. Gene filtering was also performed. Genes with zero counts in all cells were removed. A further filtering step kept only genes that were expressed at a threshold of ten counts detected in at least three cells. The threshold of ten counts was chosen based upon the distribution of expression levels for all genes. The fourth quartile (genes falling within the top 25% expression levels) was chosen for further analysis (Fig. S2*A*). This resulted in an average of 8475 genes expressed per cell library, with a range of 5804 to 11,207 genes (Fig. S2*B*). Counts for all remaining genes were then normalized using a library size scaling factor (computeSumFactors function in scran; Fig. S3) and subsequently converted to log_2_ counts per million (CPM) values, which were then used for downstream analysis.

Cells were then clustered based on similarities in their gene expression profiles using an unsupervised hierarchical clustering workflow (38). The tdTomato transcript was removed prior to clustering. First, the mean variance was fitted to the mean log expression using LOESS (locally weighted scatter plot smoother) regression. Genes with variance deviating more than twofold from the fit with a FDR of less than 5% were selected (38) (Fig. S4). These genes were then additionally filtered using a Spearman’s correlation test with FDR ≤0.001 and absolute value of rho >0.4 to remove noisy genes and allow for selection of highly variable genes that correlate in expression values across cells (38). This subset of genes was used for initial hierarchical clustering analysis using Ward’s distance (38) with a minimum cell cluster size of five cells. Each gene’s expression value is displayed as the log_2_-transformed CPM expression value subtracted from the median CPM for all cells for that gene (Fig. S5). Using the initial cell clusters, pairwise orthogonal contrasts were initialized to identify genes whose expression level was significantly different between the two clusters in comparison (≥twofold difference between any two clusters with FDR ≤ 0.05). An additional criterion was placed that the gene must be detected at ≥5 CPM in at least 20% of the cells within a cluster to avoid clustering on noisy genes that may be outliers within subsets of cells within a cluster. The capture site images corresponding to cells expressing genes from two cell clusters at this stage were then double-checked for doublet captures, and some cells were removed from the analysis based on doublet capture that was originally overlooked. Genes identified as differentially expressed between clusters were then used to cluster the cells again in a two-step iterative process, at which point the composition of the clusters no longer changed (Fig. 1). The tdTomato CPM value was plotted for each cell as a control for the clustering approach (highest tdTomato CPM values should be expressed in cells within the neuronal clusters based upon the known specificity of the *En1-Cre;Ai9* line for labeling neurons in the MNTB) (4, 5). The Wilcoxon rank sum test with an adjusted *p*-value of ≤ 0.005 was used to indicate that the neuronal clusters had significantly higher tdTomato CPM values than the non-neuronal clusters (Fig. S6).

### Differential gene expression analysis

Transcripts were identified as preferentially expressed within a particular cluster using two separate methods. First, pairwise orthogonal contrasts were used to detect transcripts differentially expressed in one cluster compared to every other cluster as described previously. The cutoff was ≥twofold differential expression with an FDR ≤0.05 (Tables S1–S10). This method identifies any transcript which has a significant difference in expression level between the two clusters in comparison. The benefit of this analysis is that transcripts can be identified in certain pairwise comparisons that would not be identified compared to the average of all clusters (for example if a transcript is differentially expressed in two clusters, such as oligodendrocytes and astrocytes). For the second method, nonpairwise orthogonal clusters were constructed to identify differentially expressed transcripts between one cluster and the average of the other clusters. In this case, the two identified neuronal clusters were combined into one cluster due to their similarity in the pairwise analysis (Table S1). Again, a cutoff of ≥twofold differential expression with an FDR ≤0.05 was used (Tables S11–S14).

### smFISH/IHC

smFISH experiments were performed on Cre-positive mice from the *En1-Cre;Ai9* cross at age P3 and P6. Mice were anesthetized with an i.p. injection of 2,2,2-tribromoethyl alcohol (125 mg/kg, Millipore Sigma). The mice were transcardially perfused first with filtered PBS (Millipore Sigma) at room temperature (RT), followed by filtered 4% paraformaldehyde (Thermo Fisher Scientific) in PBS at RT. Brains were rapidly dissected from the skull in ice-cold PBS and post-fixed overnight at 4 °C before cryoprotection overnight in 30% sucrose (Thermo Fisher Scientific). Brains were then embedded in tissue freezing medium (General Data Inc) and frozen at −80 °C until sectioning at 16 μm on a cryostat (Model CM3050S, Leica Biosystems).

The smFISH experiments were performed using the RNAScope Fluorescent Multiplex 2.5 Protocol according to the manufacturer’s instructions (Advanced Cell Diagnostics). A heat-induced epitope retrieval step was performed (10 mM citric acid, pH 6.0) for 15 min at 95 °C. Slides were then washed twice in PBS and subsequently in 100% ethanol (Pharmco-Aaper). Protease III (Advanced Cell Diagnostics) was added to the slides, which were placed in a hybridization oven (Model No. 241000, Boekel Scientific) for 30 min at 40 °C. Slides were then washed twice in PBS. Probe was added to the slides and hybridized for 2 h at 40 °C followed by two washes in wash buffer (Advanced Cell Diagnostics). Slides were then placed in the Amp1-4 reagents (Advanced Cell Diagnostics) for 30 min, 15 min, 30 min, and 15 min, respectively, with washing in between each amplification step in wash buffer. In all cases, probes were conjugated to Alexa 488 in the last amplification step. The probes used were mouse *Fgf9* (catalog no.: 499811; Advanced Cell Diagnostics) and *Fgfr3* (catalog no.: 440771; Advanced Cell Diagnostics).

Slides were then processed through an IHC protocol with cell type–specific antibodies. Blocking and application of primary and secondary antibodies was performed as previously described (12). The antibodies used were DsRed (1:500, catalog no.: 632496; Clontech) for neurons in the *En1-Cre;Ai9* cross and Aldh1L1 (1:500, catalog no.: 75-140; Antibodies Inc) for astrocytes (5). DsRed antibody was used in combination with Alexa 555 donkey anti-rabbit (1:500, catalog no.: A31572; Thermo Fisher Scientific) and Aldh1L1 antibody was used in combination with Alexa 647 donkey antimouse (1:500, catalog no.: 715-605-151, Jackson Immuno Research Laboratories Inc). 4′,6-diamidino-2-phenylindole (DAPI, Advanced Cell Diagnostics) was added to the slides before mounting with Fluoromount-G (Southern Biotech). Three biological replicates with three slices per replicate were performed for all smFISH experiments.

### IHC staining

The procedure for various IHC experiments is described here with antibody information. For standard IHC experiments, mice were transcardially perfused and brains post-fixed and cryoprotected as described previously for smFISH/IHC. After cryoprotection, brains were sliced at 40 μm thickness in the coronal plane on a freezing microtome (Model HM 450, Microm). For most antibody combinations, the sections were exposed to a heat-induced epitope retrieval step (10 mM citric acid, pH 6.0) for 30 min. The exception was the transgenic Notch reporter mouse experiments because epitope retrieval degraded the endogenous GFP signal, so these experiments did not utilize epitope retrieval. In most cases, slices were blocked and permeabilized with 3% donkey serum in PBS with 0.3% Triton. The exception was for the PNN labeling with brevican and hapln1 antibodies. In these experiments, block was performed with the exclusion of Triton. All antibodies were applied overnight in 3% donkey serum in PBS. On the second day, slices were washed three times for 10 min each in PBS with 0.1% Triton, followed by secondary antibody application for 2 h at RT in 3% donkey serum in PBS. Appropriate secondary antibodies were used (Jackson Immuno Research Laboratories, Inc). Slices were washed three times for 10 min each. DAPI was applied for 20 min followed by a PBS wash. Slices were then wet-mounted in PBS.

In the transgenic Notch reporter experiments (Fig. 3), endogenous GFP and Cd31 antibody labeling (1:250, catalog no.: AF3628; R&D Systems) were imaged together. Three littermate replicates were investigated at P3, P6, and P10 (three separate litters for three separate experiments). For the Cd31 developmental comparisons, C57Bl6/J brains were used. P3, P6 and P9 mice were sampled from the same litters (three separate litters for three separate experiments). Map2 antibody (1:2500, catalog no.: CPCA-Map2; EnCor Biotechnology) was used to identify the boundaries of the MNTB.

Gfap, Bcan, Hapln1, and Vglut1/2 staining were all performed in the Fgf9 cKO mice. Gfap was assessed at P6 and P14. Bcan, Hapln1, and Vglut1/2 were assessed at P14. Gfap (1:500, catalog no.: MAB360; EMD Millipore) was combined with Map2 antibody to identify the boundaries of the MNTB for Gfap measurements within the nucleus. Vglut1 (1:2500, catalog no.: AB5905; EMD Millipore) and Vglut2 (1:2500, catalog no.: AB2251-I) antibodies were used to label the CH terminal. Bcan (1:1000, catalog no.: 610894; BD Biosciences) and Hapln1 (1:400, catalog no.: AF2604; R&D Systems) antibodies were used to label the PNNs. For Gfap, Vglut1/2, Bcan, and Hapln1 labeling in the Fgf9 cKO mice, 5 to 6 pairs of WT and cKO littermates were used.

### Light microscopy imaging and quantification

#### smFISH quantification

smFISH experiments at P3 and P6 were performed on an inverted Zeiss LSM 710 confocal microscope equipped with a motorized stage using a 40× C-Apochromat/1.2 numerical aperture (NA) objectives (Zeiss Microscopy). Z-stacks were collected at 0.3 μm steps. Time-series experiments were performed on littermates, and imaging conditions were matched between slices. The MNTB was tiled and individual images were stitched together using Zen Black software (Zeiss Microscopy). For smFISH quantification, the z-stacks were collapsed in FIJI and regions of interest (ROIs) were drawn around individual neurons labeled with tdTomato reporter expression or astrocytes labeled with Aldh1L1. Since the tdTomato reporter labels neuron cell bodies as well as the dendrite, but the Aldh1L1 antibody labels the cell body with only proximal processes sufficiently, the ROI for quantification was drawn only around the main cell bodies of the neurons and astrocytes. This method avoids any bias from unequal compartmental labeling between the cell types. The astrocyte cell body identification was greatly assisted with 3D visualization in syGlass as previously described (5). After identifying the astrocyte cell body in 3D, the fluorescent signal labeling mRNA was quantified in FIJI. A threshold was set for the *in situ* probe fluorescent signal that was the same across all images within the dataset. Then the area of pixels within each ROI was collected using the “ROI Measure” tool. The pixels were analyzed on a per cell basis (greater than 500 neurons at P3 and P6; greater than 250 astrocytes at P3 and P6) and averaged across all cells within a slice. Three slices were imaged per biological replicate and three biological replicates were analyzed for each age and experimental probe set. The pixel area was compared for each probe between ages and also quantified for enrichment in neurons *versus* astrocytes. Two-way ANOVAs were used to test for statistical significance.

#### Transgenic Notch reporter mouse and vascular quantification

The transgenic Notch reporter and Cd31 imaging of blood vessels was performed on an inverted Zeiss LSM 710 confocal microscope. 20× Plan-Apochromat/0.75 NA or 40× C-Apochromat/1.2 NA objectives (Zeiss Microscopy) were used. Z-stacks were collected at 0.3 μm steps. Time-series experiments were performed on littermates, and imaging conditions were matched between slices. For the GFP quantification in the TNR mouse, z-stacks were collapsed and GFP+ signal was quantified in FIJI. A rolling ball radius of 25.0 was used for background subtraction on all images. Then the images were thresholded to the same level, and the percent area of GFP+ signal within each image was quantified. Two slices were analyzed for each replicate at P3, P6, and P10. Multiple *t* tests with Benjamini, Krieger, and Yekutieli correction (q = 1%) were used to determine statistical significance. For the Cd31 quantification, z-stacks were collapsed, and a ROI was drawn around the MNTB in FIJI software using the “Freehand” tool. The ROI was determined on each individual slice based on counterstaining with Map2 to label the principal neurons. The area of pixels in each ROI was determined using the “ROI Measure” tool as described previously in the smFISH quantification. All slices within the dataset were thresholded to the same level. The percent area of Cd31-positive pixels was determined by normalizing to the area of the MNTB in each ROI. Two slices were analyzed for each replicate at P3, P6, and P9, to match the ages at which structural analysis was performed using volume electron microscopy. Multiple *t* tests with Benjamini, Krieger, and Yekutieli correction (q = 1%) were used to determine statistical significance.

#### Astrocyte cell counts

Astrocyte cell counts were performed using Aldh1L1 antibody labeling as previously described (5). Briefly, a ROI was drawn around the MNTB nucleus using DAPI and Map2 signal. Then the ROI was imported into syGlass 3D virtual reality software (www.syglass.io) for cell counting using the manual “Cell Counter” tool. Aldh1L1+ cells were counted as a percentage of total cells in the MNTB labeled with DAPI.

#### Volumetric quantification of Gfap, PNNs, and CH

Imaging of Gfap in the Fgf9 cKO mice was performed on an inverted Leica SP8 3× STED laser confocal microscope using a Leica 20× HC PL APO/0.75 NA objective (Leica Microsystems). Z-stacks were collected at 0.3 μm steps. Scanning parameters were determined from one replicate pair and then left unchanged for all other replicates. Quantification of Gfap volume within the boundaries of the MNTB was performed by first segmenting out the MNTB nucleus using the “Freehand” draw tool in FIJI as described before to create an ROI based on Map2 staining. The ROI was drawn around the widest area of Map2 staining when scrolling through the z-stack and then duplicated onto the Gfap channel. The Gfap channel was imported into syGlass. All images were imported into syGlass with the same thresholding parameters. The Gfap volume was quantified using the syGlass masking tool. Two slices were quantified for each biological replicate, and six pairs of WT and KO littermate replicates were quantified at P6 and P14. All analysis was performed blinded to genotype. Unpaired *t* tests were used to determine statistical significance. The same analysis was repeated for the LSO.

Vglut1/2 labeling was used as a proxy for CH volume. Bcan and Hapln1 labeling were used as readouts for PNN volume. These datasets were imaged on an inverted Leica SP8 3× STED laser confocal microscope using a 63× HC PL APO/1.4 NA oil immersion objective (Leica Microsystems) with 1.5 digital zoom. Images were imported into syGlass and the ROI tool was used to label eight calyces or PNNs per slice. The calyces and PNNs were chosen if they were determined to be completely contained in the image volume. On average, 8 to 12 calyces or PNNs were fully contained within the image volumes, so eight ROIs were used per slice to keep the number of calyces and PNNs consistent between slices. The masking tool was then used to assess the volume of Vglut1/2, Bcan, or Hapln1 within the ROI. All images within a dataset were imported into syGlass with the same thresholding parameters. Two slices were imaged per biological replicate at P14 that contained eight ROIs each for a total of 16 ROIs per replicate. The 16 ROIs were then averaged for each replicate. Five to six WT and KO replicate pairs were analyzed for Vglut1/2, Bcan, and Hapln1. All analysis was performed blinded to genotype. Unpaired *t* tests were used to determine statistical significance.

### Segmentation and reconstruction of blood vessels from SBEM volumes

The preparation and acquisition of SBEM image volumes from FVB mouse pups at P3, P6, and P9 has been detailed previously (3). Segmentation of blood vessels from SBEM image volumes was accomplished using Seg3D software (Scientific Computing and Imaging Institute, University of Utah). Acquired image volumes for the three ages (P3, P6, and P9) were converted from Tag Image File Format image stacks into a pyramid tiff structure using the Seg3D executable “CreateLargeVolume.exe.” Image data were then opened in the Seg3D software and downsampled by a factor of 32 to enhance speed and accuracy of segmentation. Blood vessels were identified by their regular circularity, light color, and presence of surrounding endothelial cells. A high-pass filter was applied to the data using the “Threshold” tool to extract high intensity pixels, and blood vessels were further extracted by manually seeding each vessel and extracting it using the “Connected Components” tool. The resulting segmentation was checked for errors, corrected using the “Paint Brush” and “Fill Holes” tools, rendered in 3D using the “Isosurface” tool, and exported in visualization toolkit format. A custom python script was used to convert the visualization toolkit models to Object File format. Blood vessel reconstructions were then imported into the Rhinoceros CAD software (RhinoCAD, McNeel), scaled using their respective pixel dimensions (3), and their volumes were measured using the “Volume” command. Percentage of total volume was then calculated by dividing the blood vessel volume by the total image volume and multiplying by 100.

### Software for figure generation

Figures were generated using Zen Black 2.1 (Zeiss Microscopy), syGlass, Adobe Photoshop, Adobe Illustrator (Adobe Inc), MATLAB (The MathWorks, Inc), ImageJ (National Institutes of Health), and RStudio software.

## Data availability

The scRNA-Seq data is available in the SRA database under Accession #PRJNA672683.

## Supporting information

This article contains supporting information.

## Conflict of interest

G. A. S. has a financial interest in syGlass software that was used in data analysis. Other authors declare that they have no conflicts of interest with the contents of this article.
