# Supplementary material for: Transcriptional profiling reveals roles of intercellular Fgf9 signaling in astrocyte maturation and synaptic refinement during brainstem development
Source: J Biol Chem. 2022 Jun 23;298(8):102176. doi: 10.1016/j.jbc.2022.102176 (PMC9304775; doi:10.1016/j.jbc.2022.102176)
Supplement: Supplemental Figures S1–S12 [file mmc1.docx]

**Supporting Information:**

**Transcriptional profiling reveals roles of intercellular Fgf9 signaling in astrocyte maturation and synaptic refinement during brainstem development**

Ashley N. Brandebura^1,2,3^, Douglas R. Kolson^3,4,5^, Emily M. Amick^6^, Jad Ramadan^3,4^, Matthew C. Kersting^6^, Robert H. Nichol^6^, Paul S. Holcomb^3,4^, Peter H. Mathers^2,3,4,5*^, Peter Stoilov^2*^, George A. Spirou^6*^

*^1^Graduate program in Biochemistry and Molecular Biology, ^2^Department of Biochemistry, ^3^Rockefeller Neuroscience Institute ^4^Department of Otolaryngology HNS, ^5^Department of Ophthalmology, West Virginia University, Morgantown, WV, United States of America; ^6^Department of Medical Engineering, University of South Florida, Tampa, FL, United States of America*

*Present address: Ashley N. Brandebura is now at Salk Institute for Biological Studies, La Jolla, CA, United States of America*

* Co-Corresponding authors:

gspirou@usf.edu (GS)

pstoilov@hsc.wvu.edu (PS)

pmathers@hsc.wvu.edu (PHM)

The Supporting Information file contains supporting figures with legends, as well as legends for the supporting tables supplied separately in Excel format.


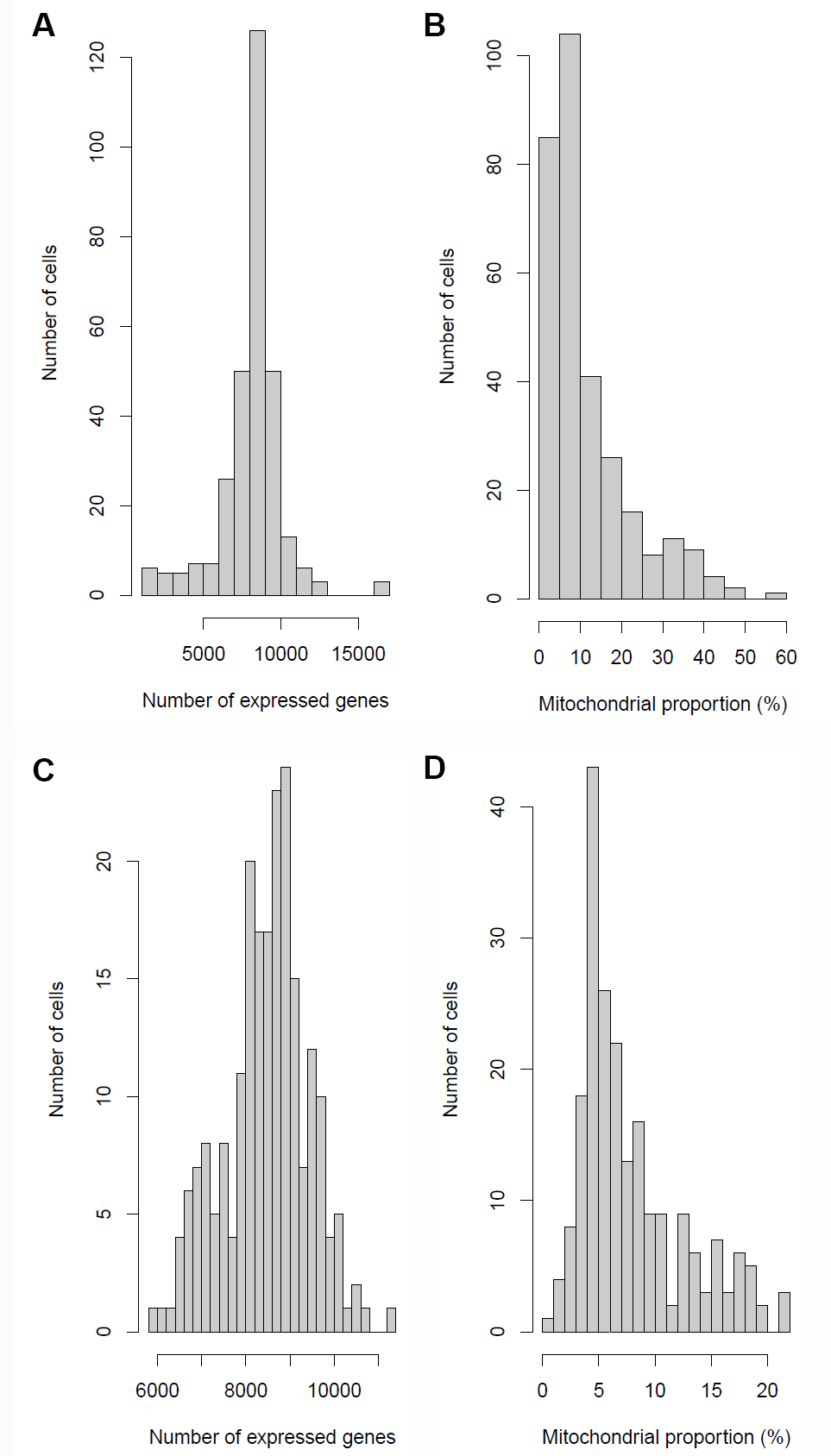


**Supporting Figure 1.** **Quality control for cells.** **A.)** Histogram displaying number of expressed genes per cell library before filtering out cells detected as outliers. **B.)** Histogram displaying percentage of reads mapping to mitochondrial transcripts before filtering out cells detected as outliers. **C.)** Histogram displaying number of expressed genes per cell library after filtering out cells detected as outliers. **D.)** Histogram displaying percentage of reads mapping to mitochondrial transcripts after filtering out cells detected as outliers. Cell were detected as outliers if ≤/≥ 3 mean absolute deviations (mad’s) outside of distribution for number of genes expressed or ≥ 3 mad’s outside of distribution for percentage of reads mapping to mitochondrial transcripts.


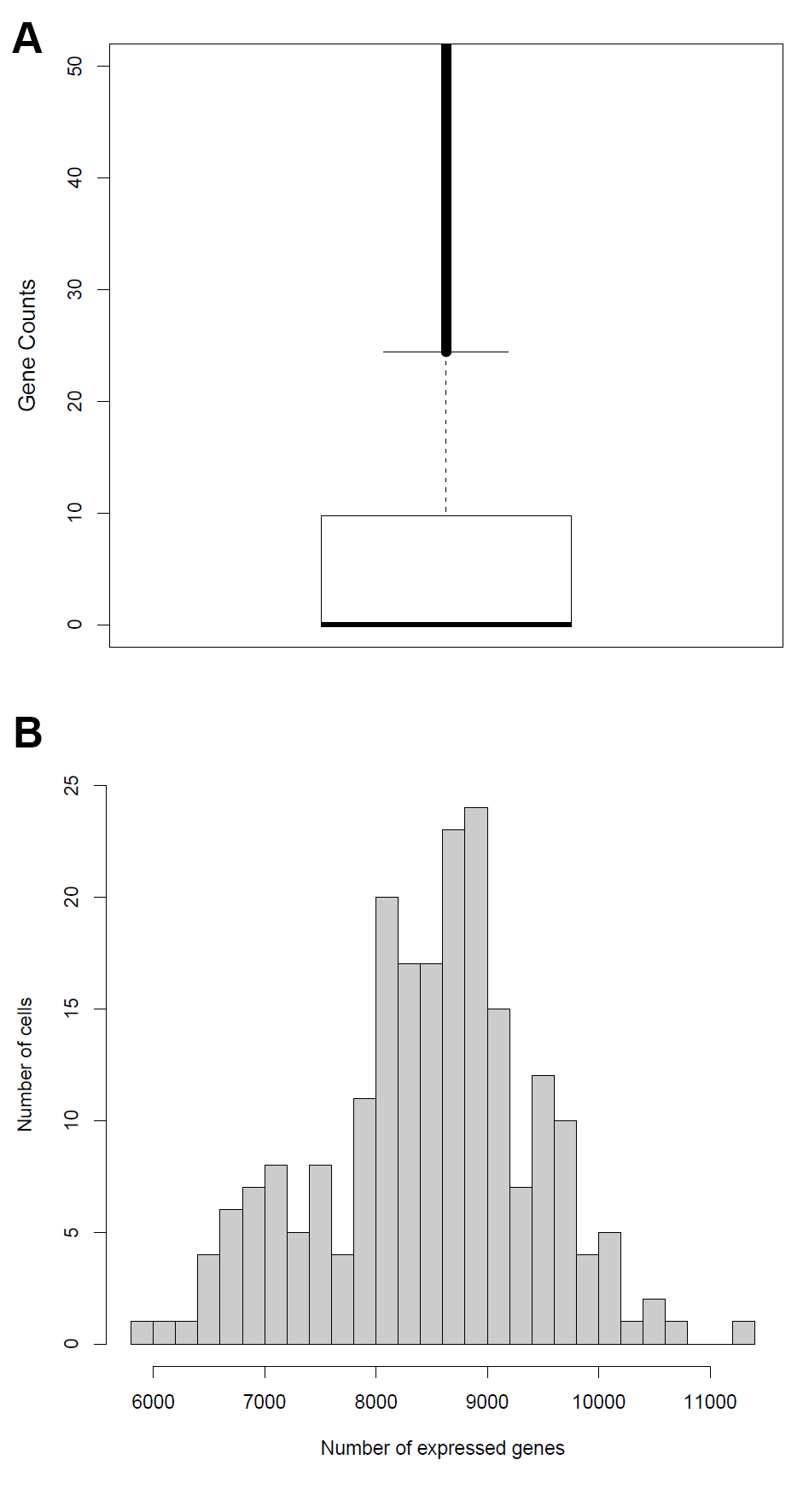


**Supporting Figure 2.** **Gene filtering metrics.** **A.)** Boxplot displaying the distribution of gene counts. Counts range from 0-99,969 counts, but the plot was set to 50 on y-axis to clearly see the median and 3^rd^ quartile. The 3^rd^ quartile value was 9.8 and thus the limit for gene detection was set to 10 in order to utilize the genes expressed in the top 25% of expression range for clustering. **B.)** Histogram displaying the number of expressed genes per cell library after gene filtering.


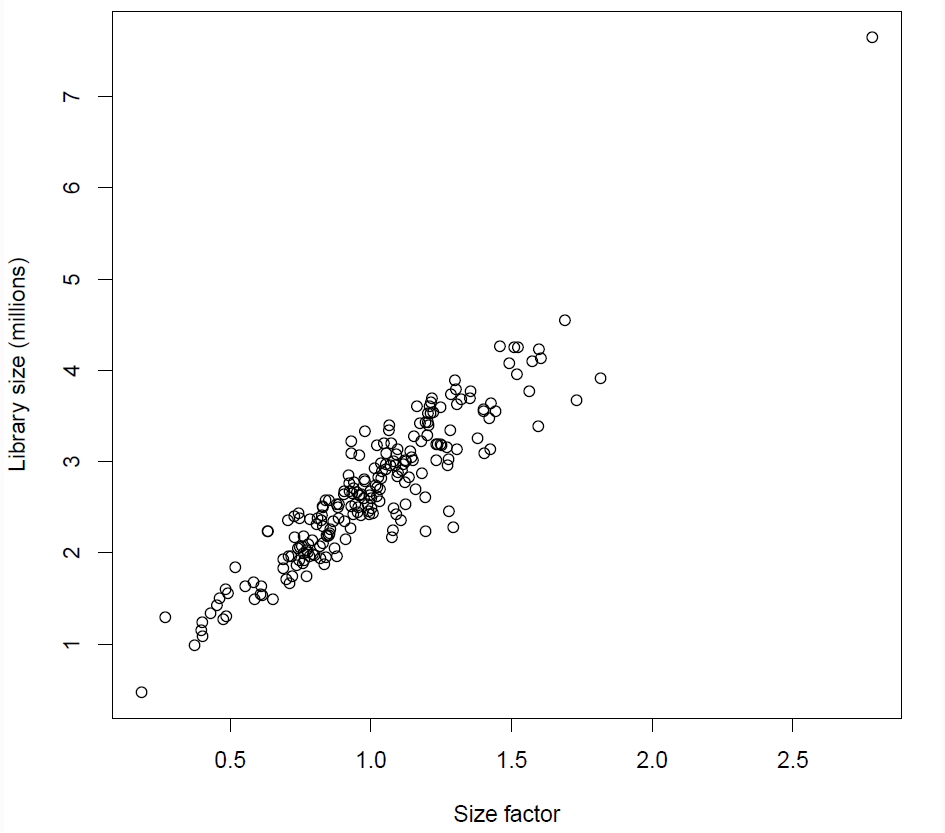


**Supporting Figure 3.** **Library size normalization.** Individual cell libraries were assigned a Size Factor to normalize for differences in library size.


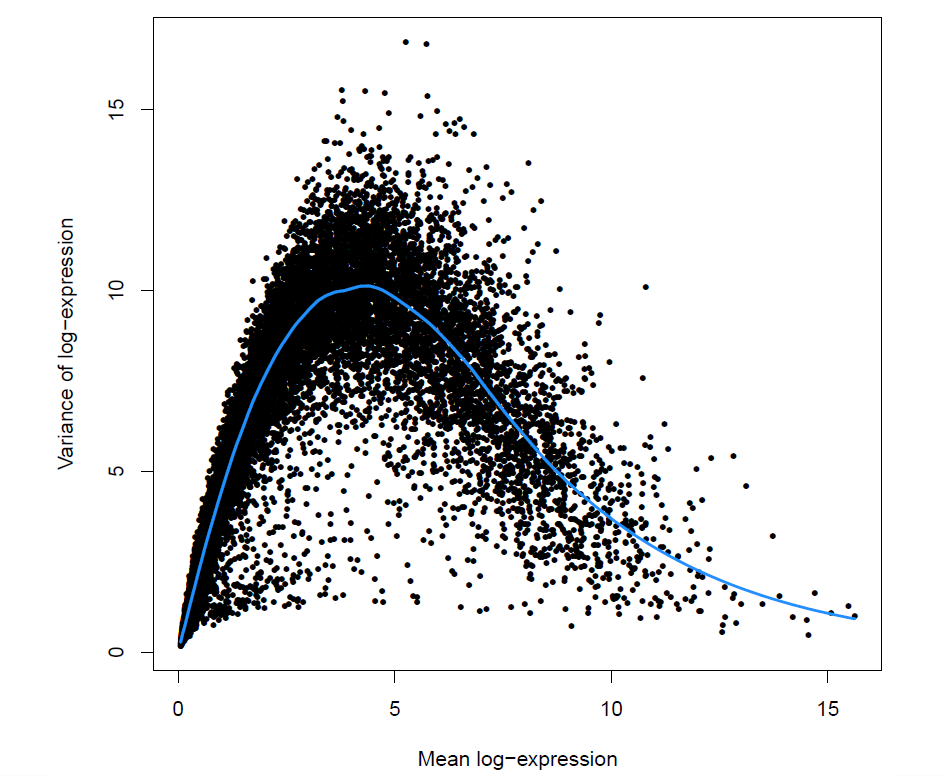


**Supporting Figure 4.** **Identification of Highly Variable Genes (HVGs).** Genes were graphed by mean log-expression (x-axis) and variance of log-expression (y-axis). A best-fit curve was generated to fit the data and HVGs were identified as being ≥ 2-fold outside of the fit with a FDR ≤ 0.05. HVGs were then used for initial round of cell clustering.


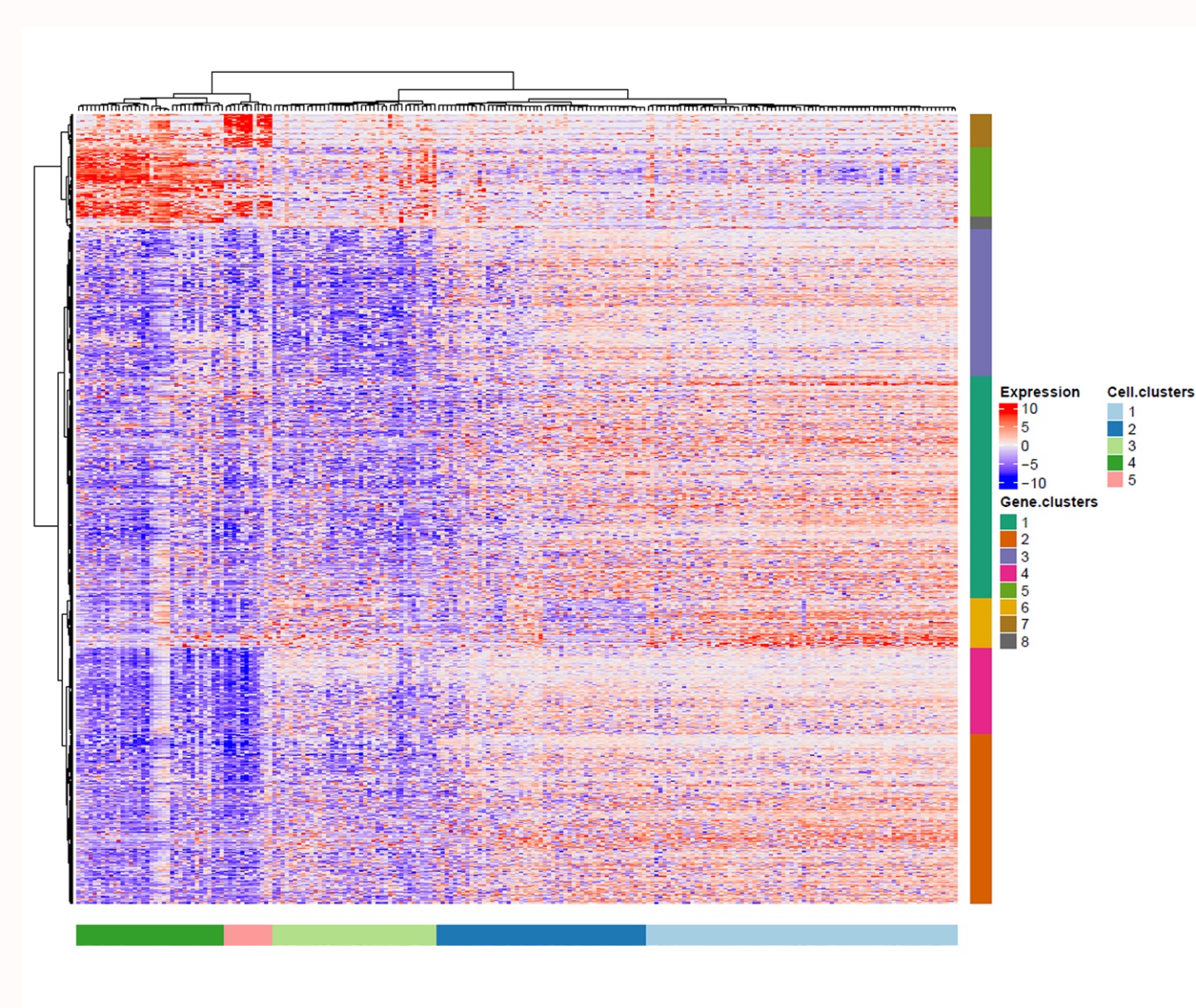


**Supporting Figure 5.** **Hierarchical clustering of cells using HVGs.** Heatmap shows expression levels of HVGs selected for initial clustering (blue to red = low to high expression). Expression values are depicted as the difference from the median (in Counts Per Million (CPM)) for any particular gene and is displayed on the log_2_ fold-change scale. The dendrogram across the top columns represents Ward’s distance for each cell (n = 215) and the dendrogram along the side represents Ward’s distance for each gene (n = 1,515). The clustering analysis yielded 8 genes clusters (right side) and 5 cell clusters (bottom).


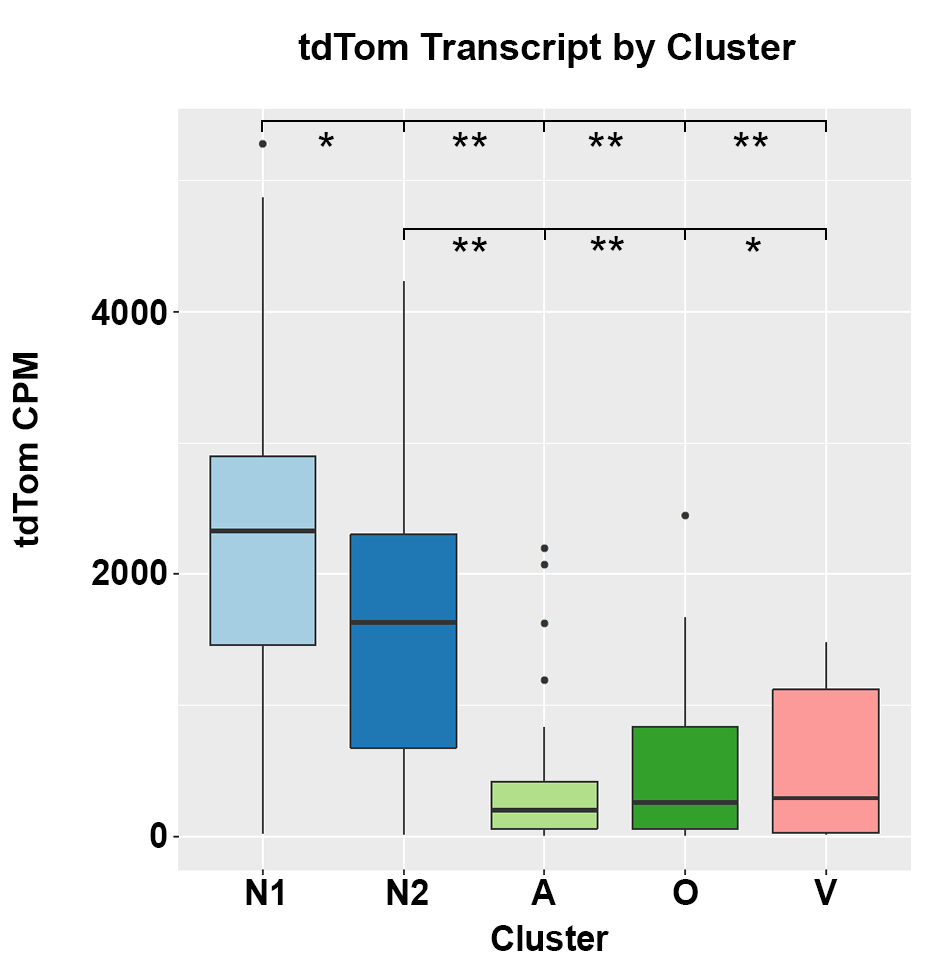


**Supporting Figure 6.** **tdTomato transcript level by cluster.** Boxplots of tdTomato transcript CPM values for each cluster. The neuronal clusters (N1 and N2) have significantly higher tdTomato levels than the nonneuronal clusters (A, O and V). N1 = Neuron Cluster 1; N2 = Neuron Cluster 2; A = Astrocyte; O = Oligodendrocyte; V = Vascular Associated Cell. * p-value ≤ 0.005; ** p-value ≤ 0.001. Wilcoxon Rank Sum Test.


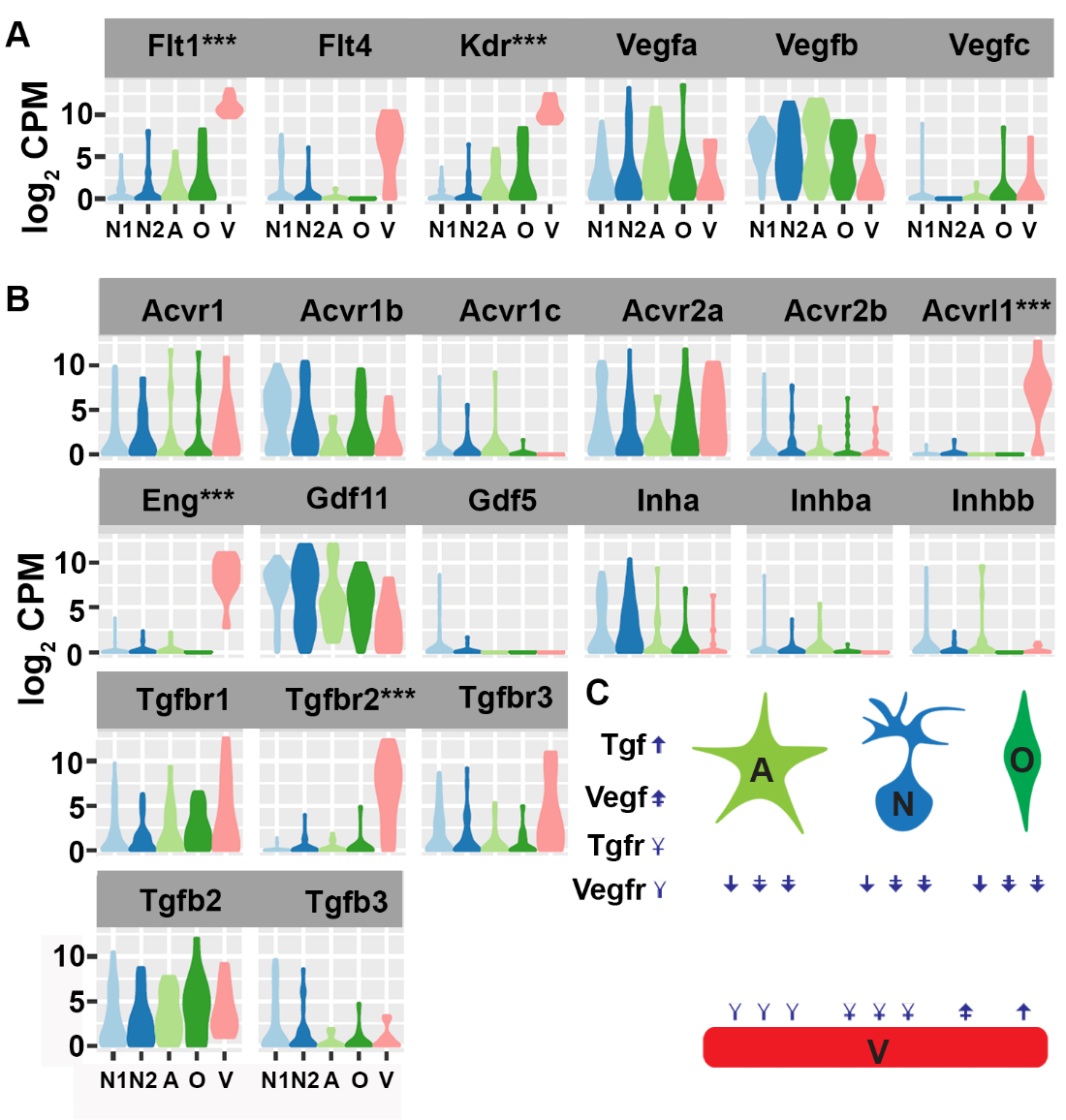


**Supporting Figure 7.** **VEGF and TGFβ ligands from neurons and glial cells transduce signaling to VACs.** **A.)** Violin plots show expression levels of transcripts which are part of the VEGF signaling pathway within each cell cluster. **B.)** Violin plots show expression levels of transcripts which are part of the TGFβ signaling pathway within each cell cluster. Expression values are displayed as the log_2_ transformed CPM. Asterisks denote genes differentially expressed in a cell cluster. *** p-value ≤ 0.001. DGE values were calculated as preferentially expressed in one cluster compared to the average of all other clusters, where N1 and N2 clusters were combined. N1 = Neuron Cluster 1; N2 = Neuron Cluster 2; A = Astrocyte; O = Oligodendrocyte; V = Vascular Associated Cell. **C.)** Schematic of expression patterns for transcripts encoding for VEGF and TGFβ ligands and receptors demonstrates that all cell types express the VEGF and TGFβ ligand transcripts while the receptor transcripts are concentrated in the VACs (V).


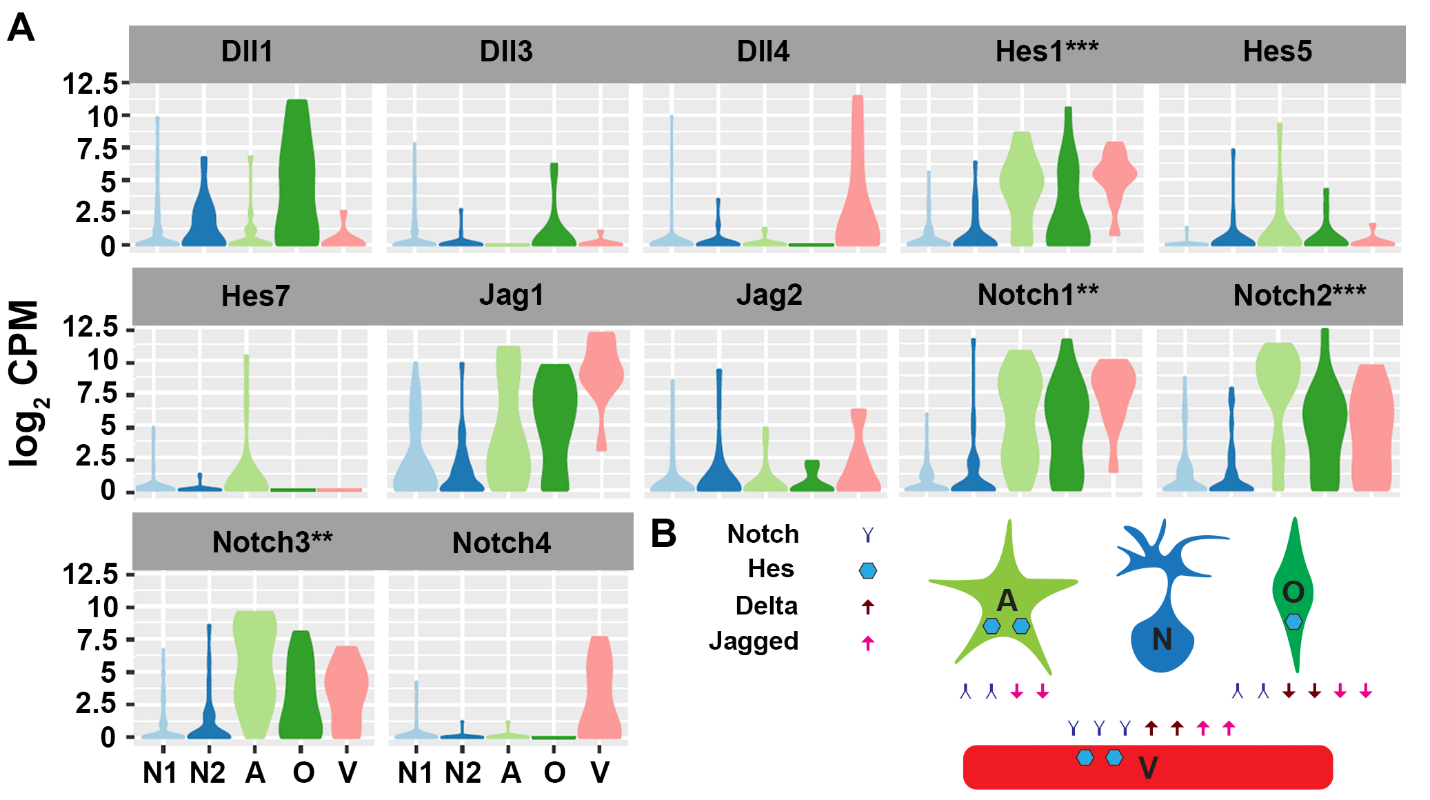


**Supporting Figure 8.** **Components of Delta-Notch signaling spread among multiple cell types.** **A.)** Violin plots show expression levels of transcripts which are part of the Delta-Notch signaling pathway within each cell cluster. Expression values are displayed as the log_2_ transformed CPM. Asterisks denote genes differentially expressed in a cell cluster. *** p-value ≤ 0.001; ** p-value ≤ 0.005. DGE values were calculated as preferentially expressed in one cluster compared to the average of all other clusters, where N1 and N2 clusters were combined. N1 = Neuron Cluster 1; N2 = Neuron Cluster 2; A = Astrocyte; O = Oligodendrocyte; V = Vascular Associated Cell. **B.)** Astrocytes express transcripts for Jag ligand, while oligodendrocytes and VACs express transcripts for both Jag and Dll ligands. The Notch receptor transcripts are expressed in astrocytes, oligodendrocytes and VACs. The Hes transcripts (transcribed downstream of active Delta-Notch signaling) are detected in astrocytes, oligodendrocytes and VACs, indicating a complex signaling pattern.


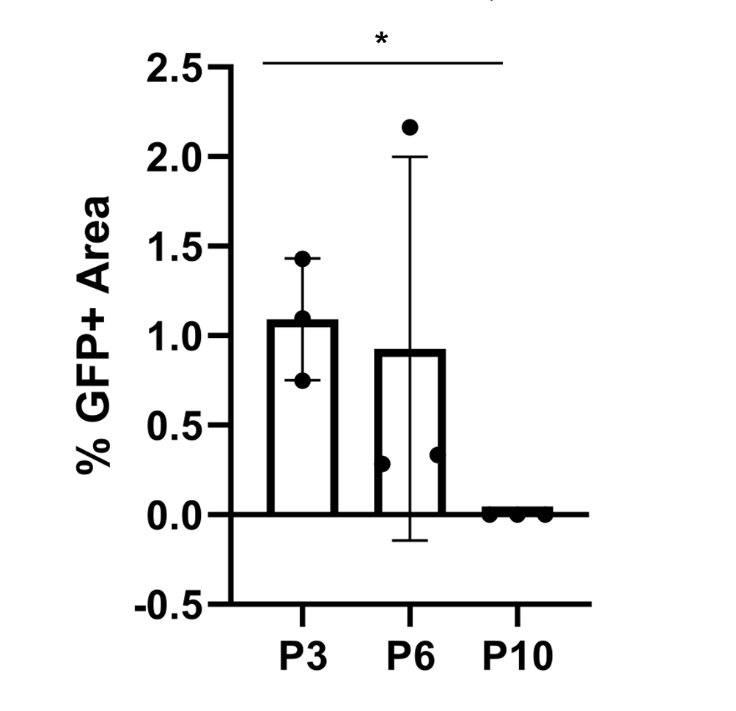


**Supporting Figure 9. Developmental Delta-Notch signal transduction quantification.** The percent area of GFP+ signal in the MNTB of TNR mice was quantified at P3, P6 and P10. The GFP+ total area significantly decreases from P3 to P10 (mean at P3 = 1.1 ± 0.34%, mean at P6 = 0.93 ± 1.1%, mean at P10 = 0.0 ± 0.0%, q-value = 0.82 for P3 vs. P6, *q-value = 0.0052 for P3 vs. P10, q-value = 0.21 for P6 vs. P10, multiple t-tests with Benjamini, Krieger and Yekutieli correction, q = 1%, n = 3 at each age). Error bars represent standard deviation.


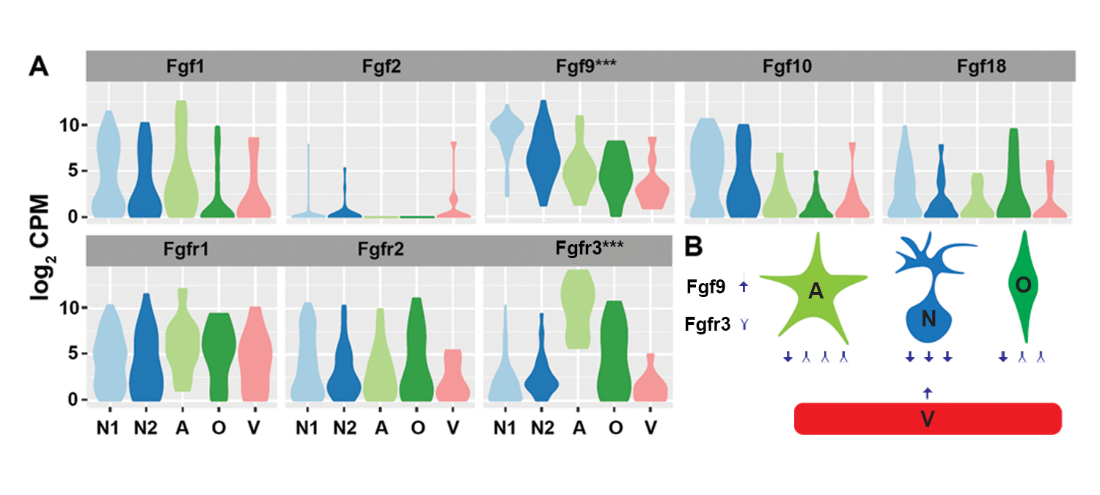


**Supporting Figure 10.** **FGF signaling pathway.** **A.)** Violin plots show expression levels of transcripts which are part of the FGF signaling pathway within each cell cluster. Expression values are displayed as the log_2_ transformed CPM. Asterisks denote genes differentially expressed in a cell cluster. *** p-value ≤ 0.001. DGE values were calculated as preferentially expressed in one cluster compared to the average of all other clusters, where N1 and N2 clusters were combined. N1 = Neuron Cluster 1; N2 = Neuron Cluster 2; A = Astrocyte; O = Oligodendrocyte; V = Vascular Associated Cell. **B.)** Schematic of expression patterns for transcripts encoding for *Fgf9* ligand and *Fgfr3* receptor demonstrates that *Fgf9* is expressed at the highest levels in neurons (N) and *Fgfr3* is expressed at the highest levels in astrocytes (A).


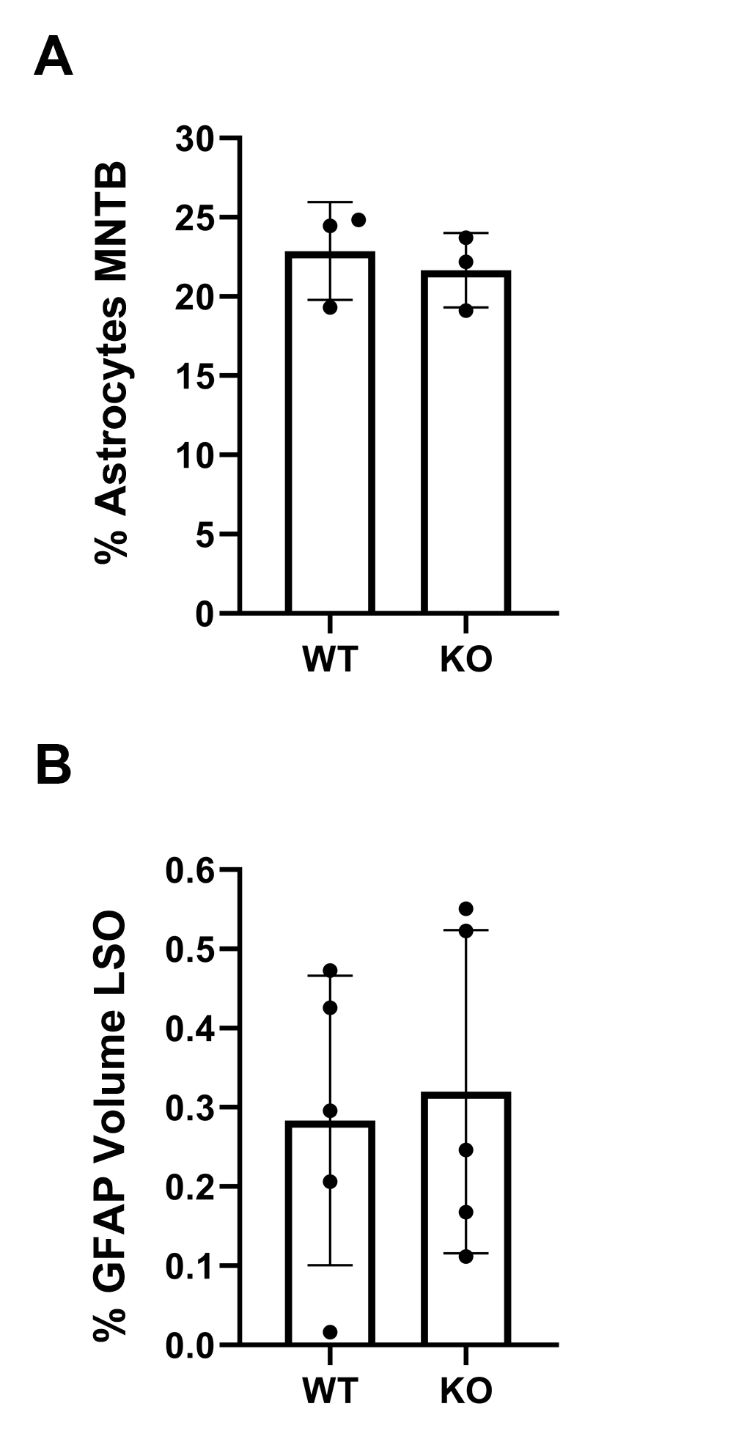


**Supporting Figure 11. No differences observed in astrocyte numbers in MNTB and increased GFAP expression is not observed in *En1*-negative anatomical nuclei. A.)** Cell counts in the MNTB using Aldh1L1 staining demonstrate that there is no change in the percentage of astrocytes in the MNTB between genotypes. **B.)** Quantification of GFAP expression in the *En1*-negative LSO nucleus shows no difference between genotypes.


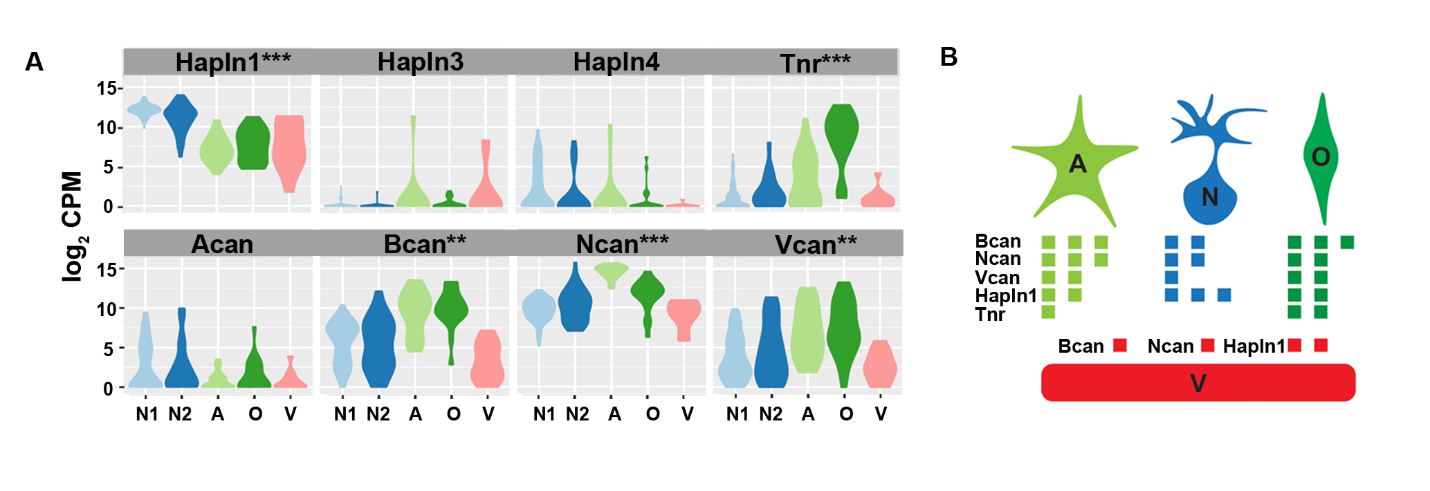


**Supporting Figure 12.** **Transcripts encoding for PNN components are distributed amongst neurons and glial cells.** **A.)** Violin plots for PNN-associated transcripts and their expression levels across different cell types. Expression values are displayed as the log_2_ transformed CPM. Asterisks denote genes significantly differentially expressed in a cell cluster. *** p-value ≤ 0.001; ** p-value ≤ 0.005. DGE values were calculated as preferentially expressed in one cluster compared to the average of all other clusters, where N1 and N2 clusters were combined. N1 = Neuron Cluster 1; N2 = Neuron Cluster 2; A = Astrocyte; O = Oligodendrocyte; V = Vascular Associated Cell. **B.)** Schematic of cell type-specific enrichment patterns of PNN-associated transcripts in the MNTB. Neurons (N) express the highest levels of *Hapln1*. Astrocytes (A) and oligodendrocytes (O) express the highest levels of CSPG transcripts (*Bcan*, *Ncan* and *Vcan*). Oligodendrocytes express the highest levels of *Tnr*. The VACs (V) express only low levels of each component.

**Supporting Table 1.** **Differentially expressed genes in N1 to N2 pairwise comparison.** Genes differentially expressed in N1 compared to N2 (left) and genes differentially expressed in N2 compared to N1 (right) with fold change (log_2_), p-value and FDR. FDR ≤ 0.05 with ≥ 2-fold change.

**Supporting Table 2.** **Differentially expressed genes in N1 to Astrocyte pairwise comparison.** Genes differentially expressed in N1 compared to Astrocytes (left) and genes differentially expressed in Astrocytes compared to N1 (right) with fold change (log_2_), p-value and FDR. FDR ≤ 0.05 with ≥ 2-fold change.

**Supporting Table 3.** **Differentially expressed genes in N1 to Oligodendrocyte pairwise comparison.** Genes differentially expressed in N1 compared to Oligodendrocytes (left) and genes differentially expressed in Oligodendrocytes compared to N1 (right) with fold change (log_2_), p-value and FDR. FDR ≤ 0.05 with ≥ 2-fold change.

**Supporting Table 4.** ***Differentially expressed genes in N1 to VAC pairwise comparison.*** Genes differentially expressed in N1 compared to VACs (left) and genes differentially expressed in VACs compared to N1 (right) with fold change (log_2_), p-value and FDR. FDR ≤ 0.05 with ≥ 2-fold change.

**Supporting Table 5.** **Differentially expressed genes in N2 to Astrocyte pairwise comparison.** Genes differentially expressed in N2 compared to Astrocytes (left) and genes differentially expressed in Astrocytes compared to N2 (right) with fold change (log_2_), p-value and FDR. FDR ≤ 0.05 with ≥ 2-fold change.

**Supporting Table 6.** **Differentially expressed genes in N2 to Oligodendrocyte pairwise comparison.** Genes differentially expressed in N2 compared to Oligodendrocytes (left) and genes differentially expressed in Oligodendrocytes compared to N2 (right) with fold change (log_2_), p-value and FDR. FDR ≤ 0.05 with ≥ 2-fold change.

**Supporting Table 7.** **Differentially expressed genes in N2 to VAC pairwise comparison.** Genes differentially expressed in N2 compared to VACs (left) and genes differentially expressed in VACs compared to N2 (right) with fold change (log_2_), p-value and FDR. FDR ≤ 0.05 with ≥ 2-fold change.

**Supporting Table 8.** **Differentially expressed genes in Astrocyte to Oligodendrocyte pairwise comparison.** Genes differentially expressed in Astrocytes compared to Oligodendrocytes (left) and genes differentially expressed in Oligodendrocytes compared to Astrocytes (right) with fold change (log_2_), p-value and FDR. FDR ≤ 0.05 with ≥ 2-fold change.

**Supporting Table 9.** **Differentially expressed genes in Astrocyte to VAC pairwise comparison.** Genes differentially expressed in Astrocytes compared to VACs (left) and genes differentially expressed in VACs compared to Astrocytes (right) with fold change (log_2_), p-value and FDR. FDR ≤ 0.05 with ≥ 2-fold change.

**Supporting Table 10.** **Differentially expressed genes in Oligodendrocyte to VAC pairwise comparison.** Genes differentially expressed in Oligodendrocytes compared to VACs (left) and genes differentially expressed in VACs compared to Oligodendrocytes (right) with fold change (log_2_), p-value and FDR. FDR ≤ 0.05 with ≥ 2-fold change.

**Supporting Table 11.** **Differentially expressed genes in neurons.** Genes differentially expressed in neurons (N1 and N2 combined) compared to the average of all nonneuronal clusters (astrocytes, oligodendrocytes and VACs) with fold change (log_2_), p-value and FDR. FDR ≤ 0.05 with ≥ 2-fold change.

**Supporting Table 12.** **Differentially expressed genes in astrocytes.** Genes differentially expressed in astrocytes compared to the average of all other clusters (N1 and N2, oligodendrocytes and VACs) with fold change (log_2_), p-value and FDR. FDR ≤ 0.05 with ≥ 2-fold change.

**Supporting Table 13. Differentially expressed genes in oligodendrocytes.** Genes differentially expressed in oligodendrocytes compared to the average of all other clusters (N1 and N2, astrocytes and VACs) with fold change (log_2_), p-value and FDR. FDR ≤ 0.05 with ≥ 2-fold change.

**Supporting Table 14.** **Differentially expressed genes in VACs.** Genes differentially expressed in VACs compared to the average of all other clusters (N1 and N2, astrocytes and oligodendrocytes) with fold change (log_2_), p-value and FDR. FDR ≤ 0.05 with ≥ 2-fold change.
